# Modeling the response of Norway spruce tree-ring carbon and oxygen isotopes to selection harvest on a drained peatland forest

**DOI:** 10.1093/treephys/tpad119

**Published:** 2023-09-26

**Authors:** Olli-Pekka Tikkasalo, Kersti Leppä, Samuli Launiainen, Mikko Peltoniemi, Raisa Mäkipää, Katja T Rinne-Garmston, Elina Sahlstedt, Giles H F Young, Aleksandra Bokareva, Annalea Lohila, Mika Korkiakoski, Pauliina Schiestl-Aalto, Aleksi Lehtonen

**Affiliations:** Natural Resources Institute Finland (LUKE), Latokartanonkaari 9, FI-00790 Helsinki, Finland; Natural Resources Institute Finland (LUKE), Latokartanonkaari 9, FI-00790 Helsinki, Finland; Natural Resources Institute Finland (LUKE), Latokartanonkaari 9, FI-00790 Helsinki, Finland; Natural Resources Institute Finland (LUKE), Latokartanonkaari 9, FI-00790 Helsinki, Finland; Natural Resources Institute Finland (LUKE), Latokartanonkaari 9, FI-00790 Helsinki, Finland; Natural Resources Institute Finland (LUKE), Latokartanonkaari 9, FI-00790 Helsinki, Finland; Natural Resources Institute Finland (LUKE), Latokartanonkaari 9, FI-00790 Helsinki, Finland; Natural Resources Institute Finland (LUKE), Latokartanonkaari 9, FI-00790 Helsinki, Finland; Natural Resources Institute Finland (LUKE), Latokartanonkaari 9, FI-00790 Helsinki, Finland; Finnish Meteorological Institute, P.O. Box 503, FI-00101 Helsinki, Finland; Institute for Atmospheric and Earth System Research (INAR)/Physics, Faculty of Sciences, University of Helsinki, P.O. Box 68, FI-00014 Helsinki, Finland; Finnish Meteorological Institute, P.O. Box 503, FI-00101 Helsinki, Finland; Institute for Atmospheric and Earth System Research (INAR)/Physics, Faculty of Sciences, University of Helsinki, P.O. Box 68, FI-00014 Helsinki, Finland; Natural Resources Institute Finland (LUKE), Latokartanonkaari 9, FI-00790 Helsinki, Finland

**Keywords:** ecosystem models, *Picea abies*

## Abstract

Continuous cover forestry (CCF) has gained interest as an alternative to even-aged management particularly on drained peatland forests. However, relatively little is known about the physiological response of suppressed trees when larger trees are removed as a part of CCF practices. Consequently, studies concentrating on process-level modeling of the response of trees to selection harvesting are also rare. Here, we compared, modeled and measured harvest response of previously suppressed Norway spruce (*Picea abies*) trees to a selection harvest. We quantified the harvest response by collecting Norway spruce tree-ring samples in a drained peatland forest site and measuring the change in stable carbon and oxygen isotopic ratios of wood formed during 2010–20, including five post-harvest years. The measured isotopic ratios were compared with ecosystem-level process model predictions for ${\kern0em }^{13}$C discrimination and ${\kern0em }^{18}$O leaf water enrichment. We found that the model predicted similar but lower harvest response than the measurements. Furthermore, accounting for mesophyll conductance was important for capturing the variation in ${\kern0em }^{13}$C discrimination. In addition, we performed sensitivity analysis on the model, which suggests that the modeled ${\kern0em }^{13}$C discrimination is sensitive to parameters related to CO_2_ transport through stomata to the mesophyll.

## Introduction

Thinning is a widely applied forest management practice to reduce competition for water, light and nutrients to enhance growth of remaining trees (Aussenac 2000). In boreal drained peatland forests, continuous cover forestry (CCF) applying regular thinnings from above (i.e., removing the dominant trees) has gained interest as an alternative to prevailing rotation forestry (Nieminen et al. 2018). Additionally, CCF practices can lead to lower short-term CO_2_ emissions compared with rotation forestry (Korkiakoski et al. 2023). The growth response of the previously suppressed trees may be delayed for several years (Koistinen and Valkonen 1993, Groot and Hökkä 2000). Thinning responses have been traditionally studied with a focus on radial growth, which, however, provides very limited insight into the physiological mechanisms underlying the growth response. Stable carbon and oxygen isotope compositions (${\delta}^{13}\mathrm{C}$ and ${\delta}^{18}\mathrm{O}$, respectively) of tree-rings reflect physiological and environmental processes at the time of tree-ring formation (McCarroll and Loader 2004) providing a complementary tool to study forest management responses and their driving mechanisms.

Tree-ring ${\delta}^{13}\mathrm{C}$ records the interplay between leaf net CO${\kern0em }_2$ assimilation (${A}_{\mathrm{n}}$) and stomatal (${g}_{\mathrm{s}}$) conductance (${A}_{\mathrm{n}}/{g}_{\mathrm{s}}$) as ${\kern0em }^{13}$C-discrimination (${\Delta}^{13}\mathrm{C}$, calculated from tree-ring ${\delta}^{13}\mathrm{C}$ and atmospheric ${\delta}^{13}\mathrm{C}$) is physiologically linked to the ratio of leaf intercellular to ambient mole fractions of CO${\kern0em }_2$ (${C}_{\mathrm{i}}/{C}_{\mathrm{a}}$) (Farquhar et al. 1989) if post-photosynthetic fractionation processes are omitted. Therefore, studies interpreting thinning-induced changes in tree-ring ${\delta}^{13}\mathrm{C}$ can identify whether increase in ${A}_{\mathrm{n}}$or ${g}_{\mathrm{s}}$ dominates the physiological response to thinning. For example, studies from sites where tree growth is water-limited typically report a more negative tree-ring ${\delta}^{13}\mathrm{C}$ (increased ${\Delta}^{13}\mathrm{C}$) as ${g}_{\mathrm{s}}$ increase relatively more than ${A}_{\mathrm{n}}$ in response to increased soil water availability after the thinning (Mcdowell et al. 2003, Giuggiola et al. 2016, Manrique-Alba et al. 2020). On light- or nutrient-limited sites, ${\delta}^{13}\mathrm{C}$ is either reported to be unaffected (Martín-Benito et al. 2010, Brooks and Mitchell 2011) or to become less negative (decrease in ${\Delta}^{13}\mathrm{C})$ (Warren et al. 2001, Powers et al. 2010) in response to thinning. In the first case, ${A}_{\mathrm{n}}$ and ${g}_{\mathrm{s}}$ are interpreted either to remain unchanged or to change such that ${A}_{\mathrm{n}}$ and ${g}_{\mathrm{s}}$ both decrease or increase proportionally. A less negative ${\delta}^{13}\mathrm{C}$ (decrease in ${\Delta}^{13}\mathrm{C}$) implies a greater relative increase in ${A}_{\mathrm{n}}$ than in ${g}_{\mathrm{s}}$, which can be attributed to changes in light conditions, increasing ${A}_{\mathrm{n}}$ more strongly than ${g}_{\mathrm{s}}$ (Di Matteo et al. 2017).

Because ${\delta}^{13}\mathrm{C}$ is influenced by two factors (${A}_{\mathrm{n}}$ and ${g}_{\mathrm{s}}$) determining the underlying changes based on ${\delta}^{13}\mathrm{C}$ alone can be challenging. To address this, a conceptual leaf-level dual-isotope model (Scheidegger et al. 2000) was developed. The model enables interpretation of ${A}_{\mathrm{n}}/{g}_{\mathrm{s}}$ variation by including tree-ring oxygen isotopes (${\delta}^{18}\mathrm{O}$). The dual-isotope model relies on changes in ${\delta}^{18}\mathrm{O}$ mainly to reflect changes in ${g}_{\mathrm{s}}$, which, however, requires a number of assumptions (Roden and Farquhar 2012, Roden and Siegwolf 2012). Among these are the requirement for a strong correlation between relative humidity (RH) and ${g}_{\mathrm{s}}$, constant source water ${\delta}^{18}\mathrm{O}$ and fairly constant ${A}_{\mathrm{n}}$. Although this can limit the applicability of the dual-isotope model, complementing ${\delta}^{13}\mathrm{C}$ data with measurements of ${\delta}^{18}\mathrm{O}$has refined the interpretation of thinning responses compared with interpretations based on ${\delta}^{13}\mathrm{C}$ alone (Powers et al. 2010, Brooks and Mitchell 2011, Giuggiola et al. 2016).

On mesic sites, such as boreal peatland forests, thinning-induced increase in growth is the result of post-harvest changes in micrometeorological conditions. While the main difference between open and closed canopies is the interception of radiation, also wind, temperature and humidity gradients within the canopy change when the forest is thinned (Aussenac 2000). All these variables have a role in driving leaf-level photosynthesis and transpiration. Multilayer soil–plant–atmosphere models, which resolve micrometeorological gradients within the canopy (Baldocchi et al. 2002, Ogée et al. 2003, Launiainen et al. 2015), have the potential to quantify the thinning-induced changes in ${A}_{\mathrm{n}}/{g}_{\mathrm{s}}$ (and thus also in ${\Delta}^{13}\mathrm{C}$) and identify the underlying mechanisms. Interpreting the changes in ${A}_{\mathrm{n}}/{g}_{\mathrm{s}}$ because of thinning may be further complemented by predictions of thinning-induced responses in leaf water ${\kern0em }^{18}$O-enrichment (${\Delta}^{18}{\mathrm{O}}_{\mathrm{lw}}$), which is driven by changes in RH, leaf temperature, transpiration and higher ${g}_{\mathrm{s}}$ relative to leaf boundary layer conductance (${g}_{\mathrm{b}})$ (Flanagan et al. 1991, Farquhar and Lloyd 1993). Despite the great potential of such models, to date, their use to support the interpretation of thinning-induced responses in tree-ring ${\delta}^{13}\mathrm{C}$ and ${\delta}^{18}\mathrm{O}$ is scarce (Giuggiola et al. 2016).

The objective of this study is to provide a process-level explanation of how the leaf-level ${\Delta}^{13}\mathrm{C}$ derived from tree-ring measurements of ${\delta}^{13}\mathrm{C}$ of Norway spruces (*Picea abies*) responds to thinning on a drained peatland forest site. To further constrain the analysis, we also derived from measurements and modeled the thinning response in ${\Delta}^{18}{\mathrm{O}}_{\mathrm{lw}}$. The observed data consisted of five spruce trees from the thinned area and another five spruce trees from a parallel intact control area. The analyzed wood was formed during the years 2010–20 including five post-thinning years. To understand the thinning-induced changes, we applied a mechanistic soil–plant–atmosphere transfer model, which resolves micrometeorological conditions in a multilayer canopy and leaf-level gas exchange (Launiainen et al. 2015, Leppä et al. 2020), in combination with leaf-level isotopic fractionation models of varying complexity (Farquhar et al. 1989, Farquhar and Lloyd 1993). Our specific objectives are to

- Analyze the observed and model-predicted ${A}_{\mathrm{n}}/{g}_{\mathrm{s}}$ changes in Norway spruce following from a selection harvest through changes in ${\Delta}^{13}\mathrm{C}$ and ${\Delta}^{18}{\mathrm{O}}_{\mathrm{lw}}$.
- Identify the main environmental drivers responsible for the observed changes in tree physiology after the selection harvest.
- Quantify the sensitivity of ${\Delta}^{13}\mathrm{C}$ and therefore of ${A}_{\mathrm{n}}/{g}_{\mathrm{s}}$ to key model parameters.

We hypothesize that (1) the model is able to predict the observed changes in ${\Delta}^{13}\mathrm{C}$ and ${A}_{\mathrm{n}}/{g}_{\mathrm{s}}$ resulting from the selection harvest but (2) the changes in ${\Delta}^{13}\mathrm{C}$ caused by the selection harvest cannot be predicted with the most simple ${\Delta}^{13}\mathrm{C}$ model which does not explicitly account for mesophyll conductance, (3) tree removal increases the availability of photosynthetically active radiation (PAR) which increases ${A}_{\mathrm{n}}$ more than ${g}_{\mathrm{s}}$ since water is not limiting CO${\kern0em }_2$ assimilation on the sampled peatland site and (4) after the selection harvest, the ${A}_{\mathrm{n}}/{g}_{\mathrm{s}}$ ratio is the most sensitive to parameters that determine the maximum CO${\kern0em }_2$ assimilation rate.

## Materials and methods

### Study site and measurements

#### Site description

The study site is a fertile peatland forest Lettosuo in Southern Finland (60°38^′^N, 23°57^′^E; Korkiakoski et al. 2020, Leppä et al. 2020). The site was originally drained with widely spaced, manually dug ditches in the 1930s, and later (in 1969) more intensively with 1-m-deep ditches spaced ca 45 m apart. The long-term (1981–2010) annual mean temperature and precipitation in the area are 4.6 °C and 627 mm, respectively (Pirinen et al. 2012). Snow typically covers the ground from December to April. More details on the study site can be found in Linkosalmi et al. (2015), Bhuiyan et al. (2017), Korkiakoski et al. (2017, 2020) and Leppä et al. (2020).

Before thinning in March 2016, the two-storied tree stand consisted of a mixture of Scots pine (*Pinus sylvestris*) and pubescent birch (*Betula pubescens*) in the dominant layer, with a dense undergrowth of Norway spruce (*Picea abies*). During thinning, mostly pines and some of the large birches and spruces were harvested reducing the stand basal area from 32 to 10 m${\kern0em }^2$ ha${\kern0em }^{-1}$ (Leppä et al. 2020). Part of the area was left intact as a control.

#### Tree core sampling and characteristics of surrounding stands

In October 2020, five suppressed Norway spruce trees (target trees) from both the thinned and the control areas were cored for tree-ring isotopic analysis. The diameter at breast height (DBH, at the height of 1.3 m) of the target trees varied between 7.9 and 15 cm, whereas their height was 6.6–12.3 m. Crown base height of the target trees was also measured.

For the 10 target trees, the surrounding stand within 5.64 m radius (i.e., 100 m${\kern0em }^2$ circular plot) was characterized by identifying tree species, and by recording DBH or stump diameter of standing trees and harvested trees, respectively. The DBH ($D$, cm) of harvested trees was calculated based on stump diameter (${D}_{\mathrm{s}}$, cm) following Laasasenaho (1982):


(1) \begin{equation*} D=\left({D}_{\mathrm{s}}-2\right)/1.25 \end{equation*}

For modeling purposes, leaf area index (LAI, m${\kern0em }^2$ m${\kern0em }^{-2}$) and leaf area density (LAD, m${\kern0em }^2$ m${\kern0em }^{-3}$) profiles for pine, spruce and birch were estimated for pre- and post-thinning conditions for each 100 m${\kern0em }^2$ plot. First, tree height and crown base height of all trees on the 100 m^2^ plots were estimated for each measured tree using the species-specific functions (Leppä et al. 2020). Next, leaf area (m${\kern0em }^2$) for each tree was derived based on DBH, crown base height and tree height using foliage biomass functions for pine, spruce and birch (Tupek et al. 2015, Lehtonen et al. 2020) and their specific leaf area values (Härkönen et al. 2015). The vertical distribution of leaf area of each tree was derived based on Tahvanainen and Forss (2008). Finally, species-specific LAI and LAD for each plot were obtained as the sum of the values for individual trees of that species within the plot divided by the area of the plot (Figure S1 available as Supplementary data at *Tree Physiology Online*). LAI was on average 5.8 m${\kern0em }^2$ m${\kern0em }^{-2}$ (range 4.8–7.1), 5.3 m${\kern0em }^2$ m${\kern0em }^{-2}$ (4.2–6.2) and 2.6 m${\kern0em }^2$ m${\kern0em }^{-2}$ (1.3–3.8) for the control plots, the harvested plots before thinning and the harvested plots after thinning, respectively.

#### Sample preparation and isotopic analysis

We measured the intra-annual tree-ring ${\delta}^{13}\mathrm{C}$ with laser ablation isotope ratio mass spectrometry (LA-IRMS) at the Stable Isotope Laboratory of Luke. The tree-ring ${\delta}^{13}\mathrm{C}$ data in our study are the same as in Lehtonen et al. (2023). The data are comprised of typically 10 evenly distributed ${\delta}^{13}\mathrm{C}$ data points per annual ring extending from 2010 to 2020. For the present study, the resin extracted cores used for ${\delta}^{13}\mathrm{C}$ analysis in Lehtonen et al. (2023) were prepared for ${\delta}^{18}\mathrm{O}$ analysis as follows. For the years 2010–20, early wood (EW) and late wood (LW) were separated under binocular microscope using a scalpel. The resulting wood subsamples were homogenized and weighed into silver capsules for ${\delta}^{18}\mathrm{O}$ analysis, which was conducted using a high temperature elemental analysis isotope mass spectrometry (HT-EA-IRMS, instrument by Sercon Ltd, Crewe, Cheshire, United Kingdom). Resulting data were normalized using reference materials with varying δ^18^O values: IAEA-601 (+23.14‰), lactose (+21.05‰) and sucrose (+36.62‰), which were run concurrently with the samples. Analytical precision was $\pm$0.14‰, based on replicate analysis (*n* = 12) of a quality control material. For subsequent model-measurement comparisons, the stand-presentative average EW and LW *δ*^18^O values were calculated for each year 2010–20 from the five cores (i.e., five separate trees) per treatment (Leavitt and Long 1984), with $\pm$0.2–1.7‰ standard deviation.

#### *Formation time of measured* ${\delta}^{13}C$ *and* ${\delta}^{18}O$

To determine the formation time of the xylem cells that represent each measured ${\delta}^{13}\mathrm{C}$ value, we first determined the EW and LW formation periods for each year with the CASSIA model that predicts growth timing based on environmental factors and internal growth control (Schiestl-Aalto et al. 2015). CASSIA was parameterized for Norway spruce and used for this same site by Lehtonen et al. (2023). The maturation time of the first EW cells was set to 24 days after the predicted onset of tracheid formation of the CASSIA model (Schiestl-Aalto, unpublished data). The 24-day delay corresponds to the time of cell formation and is based on Norway spruce microcore measurements at Station for Measuring Forest Ecosystem–Atmosphere Relations (SMEAR) II in Hyytiälä Southern Finland during 2007–09. For each ${\delta}^{13}\mathrm{C}$ measurement, we determined visually whether it belonged to EW or LW. We obtained the formation time for each measurement by dividing the ${\delta}^{13}\mathrm{C}$ datapoints equally in time inside their respective EW and LW periods defined by CASSIA model. For ${\delta}^{18}\mathrm{O}$, we had one measurement representing EW and one for LW.

#### *Conversion of measured* ${\delta}^{13}C$ *and* ${\delta}^{18}O$ *to* ${\Delta}^{13}C$ *and* ${\Delta}^{18}{O}_{lw}$

The measured tree-ring ${\delta}^{13}\mathrm{C}$ values will be hereafter expressed as leaf-level photosynthetic discrimination against ^13^C during carbon fixation (Farquhar et al. 1989):


(2) \begin{equation*} {\Delta}^{13}\mathrm{C}=\frac{\delta^{13}{\mathrm{C}}_{\mathrm{atm}}-{\delta}^{13}{\mathrm{C}}_{\mathrm{wood}}}{1+{\delta}^{13}{\mathrm{C}}_{\mathrm{wood}}} \end{equation*}

where ${\delta}^{13}{\mathrm{C}}_{\mathrm{wood}}$ is the observed tree-ring value and ${\delta}^{13}{\mathrm{C}}_{\mathrm{atm}}$ is the ${\delta}^{13}\mathrm{C}$ of ambient CO${\kern0em }_2$. ${\delta}^{13}{\mathrm{C}}_{\mathrm{wood}}$ was measured from resin extracted wood, which recent studies have shown to be only 0–1‰ more enriched compared with leaf sucrose (Rinne et al. 2015, Tang et al. 2022, Rinne-Garmston et al. 2023). Therefore, we chose not to include an offset correction in Eq. (2) as was done for oxygen (Eq. (3)). ${\delta}^{13}{\mathrm{C}}_{\mathrm{atm}}$ was available from Pallas-Sammaltunturi GAW-station at weekly resolution (White et al. 2021). ${\delta}^{13}{\mathrm{C}}_{\mathrm{atm}}$ in the lower canopy may differ between closed and open canopies (i.e., before and after the selection harvest) because of differences in turbulent mixing (Bowling et al. 1999). However, air samples collected during 2019–20 at the study site indicated a median difference between control and harvested areas of <0.3‰ (Lehtonen et al. 2023). We note that Eq. (2) neglects any post-photosynthetic fractionation processes and will address its implications in the discussion. We refer the ${\Delta}^{13}\mathrm{C}$ derived from the measured ${\delta}^{13}\mathrm{C}$ as measured ${\Delta}^{13}\mathrm{C}$ hereafter to distinguish it from the model-predicted ${\Delta}^{13}$C.

Measured ${\delta}^{18}\mathrm{O}$ values were converted to leaf water ${\kern0em }^{18}$O-enrichment over source water (Barbour and Farquhar 2000):


(3) \begin{equation*} {\Delta}^{18}{\mathrm{O}}_{\mathrm{lw}}=\frac{\Delta^{18}{\mathrm{O}}_{\mathrm{wood}}-{\epsilon}_{\mathrm{wc}}-{\epsilon}_{\mathrm{cp}}}{1-{p}_{\mathrm{ex}}{p}_{\mathrm{x}}} \end{equation*}

where ${\epsilon}_{\mathrm{wc}}$ is the biochemical fractionation factor associated with oxygen isotope exchange between carbonyl oxygen and leaf water. ${\epsilon}_{\mathrm{wc}}$ was defined as temperature-dependent (see Figure 1 in Sternberg and Ellsworth 2011), which was recently found to be critical in leaf water ${\delta}^{18}\mathrm{O}$ predictions by Hirl et al. (2021) and Leppä et al. (2022). ${\epsilon}_{\mathrm{cp}}$ (3.5‰) is the offset in enrichment between wood and cellulose (Gori et al. 2013), ${p}_{\mathrm{ex}}{p}_{\mathrm{x}}$ (0.4) reflects the proportion of oxygen in cellulose derived from source water (Gessler et al. 2014) and ${\Delta}^{18}{\mathrm{O}}_{\mathrm{wood}}$ is the ${\kern0em }^{18}$O-enrichment of wood over source water:


(4) \begin{equation*} {\Delta}^{18}{\mathrm{O}}_{\mathrm{wood}}=\frac{\delta^{18}{\mathrm{O}}_{\mathrm{wood}}-{\delta}^{18}{\mathrm{O}}_{\mathrm{s}}}{1+{\delta}^{18}{\mathrm{O}}_{\mathrm{s}}} \end{equation*}

where ${\delta}^{18}{\mathrm{O}}_{\mathrm{s}}$ is the isotopic composition of source water. ${\delta}^{18}{\mathrm{O}}_{\mathrm{s}}$ was set to −10‰ according to soil and twig water measurements at SMEAR II during tree growing seasons of 2018 and 2019 (61°51′N, 24°17′E; Leppä et al. 2022). By using the constant ${\delta}^{18}{\mathrm{O}}_{\mathrm{s}}$, we assume that the isotopic composition of precipitation and the soil water evaporation are similar at Hyytiälä and Lettosuo. Applying a constant ${\delta}^{18}{\mathrm{O}}_{\mathrm{s}}$ was supported by Leppä et al. (2022), who showed that temporal variability in leaf water ${\kern0em }^{18}$O-enrichment clearly dominated over that of ${\delta}^{18}{\mathrm{O}}_{\mathrm{s}}$. The option of using ${\delta}^{18}\mathrm{O}$ of precipitation from the isotope enabled, nudged atmospheric general circulation model IsoGSM (Yoshimura et al. 2008, 2011) as ${\delta}^{18}{\mathrm{O}}_{\mathrm{s}}$ instead of a constant ${\delta}^{18}{\mathrm{O}}_{\mathrm{s}}$ was explored, but it resulted in a significantly poorer model fit (results not shown). This is in line with Hirl et al. (2019), who found it necessary to correct the IsoGSM data with an offset and showed that it performed as inferior model input compared with on-site measured ${\delta}^{18}\mathrm{O}$ of precipitation. We further assumed there to be no difference in ${\delta}^{18}{\mathrm{O}}_{\mathrm{s}}$ between the control and the harvested areas in line with other studies on wet sites (Brooks and Mitchell 2011). The validity of this assumption will be discussed later.

#### Meteorological conditions

Meteorological variables were recorded since September 2009 as 30-min averages above the canopy at 25.5 m. The data used for modeling included air temperature (HMP45D, Vaisala Corporation), RH (HMP45D, Vaisala Corporation), atmospheric pressure (PMT16A, Vaisala Corporation), incoming global radiation (Pyranometer CMP3, Kipp & Zonen, Delft, The Netherlands), incoming PAR (PQS1 PAR Quantum sensor, Kipp & Zonen), wind speed and friction velocity (METEK USA-1, METEK GMbH), and precipitation (Casella Ltd Par No 10000E-04, measured at 6 m height). Meteorological data from three nearby weather stations (Jokioinen (60°48′N, 23°30′E), Somero (60°39′N, 23°48′E) and Salo Kiikala (60°27′N, 23°39′E)), operated by the Finnish Meteorological Institute, were used to complete the wintertime precipitation data and to fill the gaps in the on-site meteorological time series.

In the absence of on-site measurements, downwelling longwave radiation was used from a site ca 1 km northeast of the study site (Tervalamminsuo), where it has been recorded since 2013 (CGR4 Pyrgeometer, Kipp & Zonen). Before 2013 and for gaps in data, downwelling longwave radiation was estimated as in Leppä et al. (2020) following Song et al. (2009). The method proposed by Song et al. (2009) was also applied for decomposing measured global radiation into its direct and diffuse components. Finally, the ambient CO${\kern0em }_2$ mole fraction was estimated based on the yearly trend in May–September data recorded at SMEAR II during 1997–2019. Resulting yearly ambient CO${\kern0em }_2$ increased linearly from 388 to 410 p.p.m. during 2010–20.

### Modeling

#### Soil–plant–atmosphere transfer model

The pyAPES model (Launiainen et al. 2015, 2022, Leppä et al. 2020) was applied to investigate the micrometeorological conditions of the target trees before and after the selection harvest and their implications to leaf photosynthesis and ${C}_{\mathrm{i}}/{C}_{\mathrm{a}}$. The model computes energy, H${\kern0em }_2$O and CO${\kern0em }_2$ fluxes in a multilayer multispecies forest canopy accounting for variable LAD and micrometeorological gradients within the canopy. Additionally, pyAPES has submodules for a forest floor covered by mosses and/or litter, a snow pack and an underlying soil profile, whose descriptions can be found elsewhere (Launiainen et al. 2019, Leppä et al. 2020). As forcing variables, the model uses 30-min meteorological variables at a reference level above the canopy, including precipitation, downwelling longwave radiation, direct and diffuse PAR, and near-infrared radiation, wind speed, atmospheric pressure, air temperature, and ambient mole fractions of H${\kern0em }_2$O and CO${\kern0em }_2.$ In the model, the canopy is composed of vascular plant types (here pine, birch and spruce, and field layer vegetation), which are characterized by their structural and physiological properties, including LAD, photosynthetic parameters, water use traits and seasonal acclimation parameters among others. Leaf gas exchange, coupled with leaf energy balance, is solved separately for sunlit and shaded leaves of each plant type exposed to different radiation regimes at each canopy layer (here 100 layers).

Leaf net CO${\kern0em }_2$ exchange (${A}_{\mathrm{n}}$, $\mu$mol m${\kern0em }^{-2}$(leaf) s${\kern0em }^{-1}$) is computed following Farquhar et al. (1980), with the co-limitation of Rubisco- and RuBP-regeneration rates (Collatz et al. 1990), temperature-dependent mitochondrial respiration (${r}_{\mathrm{d}}$, *μ*mol m^−2^(leaf) s^−1^; Bernacchi et al. 2001) and temperature adjustments to the kinetic rate constants (Medlyn et al. 2002). Leaf-scale stomatal conductance (${g}_{\mathrm{s}}$, mol m${\kern0em }^{-2}$ (leaf) s${\kern0em }^{-1}$) for CO${\kern0em }_2$ is defined by the unified stomatal model relying on the principal that stomata act to minimize the amount of water used per unit carbon gained (Medlyn et al. 2011)


(5) \begin{equation*} {g}_{\mathrm{s}}={g}_0+\left(1+\frac{g_1}{\sqrt{D_{\mathrm{l}}}}\right)\frac{A_{\mathrm{n}}}{C_{\mathrm{s}}} \end{equation*}

where ${D}_{\mathrm{l}}$ is the leaf to air vapor pressure deficit (kPa) affected by leaf temperature, ${g}_0$ (mol m${\kern0em }^{-2}$ (leaf) s${\kern0em }^{-1}$) is residual conductance, ${g}_1$ (kPa${\kern0em }^{0.5}$) is a parameter proportional to the marginal water-use efficiency reflecting plant water use strategies and ${C}_{\mathrm{s}}$ ($\mu$mol mol${\kern0em }^{-1}$) is the CO${\kern0em }_2$ mole fraction at leaf surface. ${C}_{\mathrm{s}}$ is calculated based on ${A}_{\mathrm{n}}$, the CO${\kern0em }_2$ mole fraction in the canopy air space (${C}_{\mathrm{a}}$) and boundary layer conductance for CO${\kern0em }_2$ (*g*_b_; Launiainen et al. 2015).

The micrometeorological conditions within the canopy are solved iteratively with leaf gas exchange and energy balance. The transfer and absorption of shortwave and longwave radiation in the layered porous medium defined by the total canopy LAD distribution are computed following Zhao and Qualls (2005, 2006). Standard first-order closure schemes are applied to resolve the turbulent transport in the canopy air space and resulting vertical gradients of wind speed, air temperature, H${\kern0em }_2$O and CO${\kern0em }_2$ (Launiainen et al. 2015). Furthermore, precipitation interception within the canopy layers and wet leaf energy balance is solved following Watanabe and Mizutani (1996). The pyAPES predictions of leaf ${C}_{\mathrm{i}}/{C}_{\mathrm{a}}$, ${A}_{\mathrm{n}}$ and ${g}_{\mathrm{s}}$ in each canopy layer and plant type were used as inputs to leaf-level isotopic discrimination module.

#### Leaf-level isotope discrimination models

At leaf-level the isotopic composition of new assimilates reflects current photosynthetic conditions. According to the classical formulation of photosynthetic ${\kern0em }^{13}$C-discrimination (Farquhar et al. 1982):


(6) \begin{equation*} {\Delta}^{13}\mathrm{C}={a}_{\mathrm{b}}\frac{C_{\mathrm{a}}-{C}_{\mathrm{s}}}{C_{\mathrm{a}}}+{a}_{\mathrm{s}}\frac{C_{\mathrm{s}}-{C}_i}{C_{\mathrm{a}}}+{a}_{\mathrm{m}}\frac{C_{\mathrm{i}}-{C}_{\mathrm{c}}}{C_{\mathrm{a}}}+b\frac{C_{\mathrm{c}}}{C_{\mathrm{a}}}-f\frac{\varGamma_{\ast }}{C_{\mathrm{a}}}-e\frac{r_{\mathrm{d}}}{k{C}_{\mathrm{a}}} \end{equation*}

where ${C}_{\mathrm{a}}$, ${C}_{\mathrm{s}}$, ${C}_{\mathrm{i}}$ and ${C}_{\mathrm{c}}$ (mol mol${\kern0em }^{-1}$) are CO_2_ mole fractions in the atmosphere, at the leaf surface, in the intercellular spaces and in the chloroplasts, respectively, ${a}_{\mathrm{b}}$ (2.9‰) is the fractionation associated with diffusion through the boundary layer, ${a}_{\mathrm{s}}$ (4.4‰) the fractionation from diffusion through stomata, ${a}_{\mathrm{m}}$ (1.8‰) the fractionation because of transfer through mesophyll, $b$ (29‰) the fractionation in carboxylation, $f$ (8‰; Ghashghaie et al. 2003) the fractionation associated with photorespiration and $e$ (−6‰; Ghashghaie et al. 2003) the fractionation in mitochondrial respiration. ${\varGamma}_{\ast }$ (mol mol${\kern0em }^{-1}$) is the CO_2_ compensation point in the absence of mitochondrial respiration (Bernacchi et al. 2001) and $k$ ($=\left({A}_{\mathrm{n}}+{r}_{\mathrm{d}}\right)/\left({C}_{\mathrm{c}}-{\varGamma}_{\ast}\right)$) is the carboxylation efficiency. To predict leaf-level ${\Delta}^{13}\mathrm{C}$, we considered three approaches in applying Eq. (6): (i) reducing it to its simplest format ${\Delta}^{13}\mathrm{C}={a}_{\mathrm{s}}+\left({b}^{\prime }-{a}_{\mathrm{s}}\right){C}_{\mathrm{i}}/{C}_{\mathrm{a}}$, where ${b}^{\prime }$ is 27‰ (simple model), (ii) assuming mesophyll resistance is negligible, i.e., ${C}_{\mathrm{c}}={C}_{\mathrm{i}}$ (classical model) and (iii) using a constant mesophyll conductance to calculate ${C}_{\mathrm{c}}={C}_{\mathrm{i}}-{A}_{\mathrm{n}}/{g}_{\mathrm{m}}$ (classical model with ${g}_{\mathrm{m}}$). In the last case, ${g}_{\mathrm{m}}$ was set to 0.1 mol m${\kern0em }^{-2}$(leaf) s${\kern0em }^{-1}$, which is in the range reported earlier for coniferous species (Wingate et al. 2007, Flexas et al. 2008, Stangl et al. 2019).

Leaf water ${\kern0em }^{18}$O-enrichment was modeled assuming vapor-source water isotopic equilibrium (${\Delta}_{18}{\mathrm{O}}_{\mathrm{vapor}}=-{\epsilon}^{+}$; Ogée et al. 2003) following Farquhar and Lloyd (1993): 


(7) \begin{equation*} {\Delta}^{18}{\mathrm{O}}_{\mathrm{lw}}={f}_1\left[{\epsilon}^{+}+{\epsilon}_{\mathrm{k}}\right]\left(1-\frac{w_{\mathrm{a}}}{w_{\mathrm{i}}}\right) \end{equation*}

where ${\epsilon}^{+}$ is the (temperature-dependent) fractionation because of vapor pressure depression (Majoube 1971), ${w}_{\mathrm{a}}$ and ${w}_{\mathrm{i}}$ (mol mol${\kern0em }^{-1}$) are the mole fractions of water vapor in the atmosphere and inside the leaf, respectively and ${\epsilon}_k$ is the kinetic isotope fractionation during water vapor diffusion through stomata and leaf boundary layer: 


(8) \begin{equation*} {\epsilon}_{\mathrm{k}}=\frac{g_{\mathrm{b}}{\epsilon}_{\mathrm{k}\mathrm{s}}+{g}_{\mathrm{s}}{\epsilon}_{\mathrm{k}\mathrm{b}}}{g_{\mathrm{b}}+{g}_{\mathrm{s}}} \end{equation*}

where ${g}_{\mathrm{s}}$ and ${g}_{\mathrm{b}}$ (mol m${\kern0em }^{-2}$ (leaf) s${\kern0em }^{-1}$) are stomatal and boundary layer conductances for H${\kern0em }_2$O and ${\epsilon}_{\mathrm{ks}}$ (28‰) and ${\epsilon}_{\mathrm{kb}}$ (19‰) are the fractionation factors associated with the diffusion through stomata and the boundary layer, respectively (Merlivat 1978). To predict leaf-level ${\Delta}^{18}{\mathrm{O}}_{\mathrm{lw}}$, we applied two variations of Eq. (7): (i) the Craig–Gordon model by setting ${f}_1=1$ (Craig and Gordon 1965, Dongmann et al. 1974) and (ii) the Péclet model defining ${f}_1=\left(1-{e}^{-\wp}\right)/\wp$ (Barbour et al. 2000, 2004), where $\wp = EL/(CD)$, $L$ (m) is the effective mixing length, $E$ (mol m${\kern0em }^{-2}$ s${\kern0em }^{-1}$) the transpiration rate, $C$ (55.5 $\times$ 10${\kern0em }^3$ mol m${\kern0em }^{-3}$) the molar density of liquid water and $D$ (2.66 $\times$ 10${\kern0em }^{-9}$ m${\kern0em }^2$ s${\kern0em }^{-1}$) the diffusivity of H${\kern0em }_2^{18}$O in liquid water. $L$ was set to 15 mm in line with Leppä et al. (2022), which corresponds to 30 mm when $E$ is expressed against whole-sided leaf area instead of one-sided leaf area as here.

The leaf-level ${\Delta}^{13}\mathrm{C}$ and ${\Delta}^{18}$O${\kern0em }_{\mathrm{lw}}$ values were aggregated to tree-level to be compared against values derived from observed tree-ring isotopic signals using assimilation-based weighting and neglecting further post-photosynthetic fractionation processes: 


(9) \begin{align*} {\Delta}_{\mathrm{tree}}=&\sum_{\mathrm{i}=0}^{100}\left({\varLambda}_{\mathrm{i}}\sum_{\mathrm{j}=\mathrm{sl},\mathrm{sh}}\left({\Delta}_{i,\mathrm{j}}{f}_{\mathrm{i},\mathrm{j}}\max \left(0,{A}_{{\mathrm{n}}_{\mathrm{i},\mathrm{j}}}\right)\right)\right)\left/\vphantom{\sum_{1}}\right. \nonumber\\&\sum_{\mathrm{i}=0}^{100}\left({\varLambda}_{\mathrm{i}}\sum_{\mathrm{j}=\mathrm{sl},\mathrm{sh}}\left({f}_{\mathrm{i},\mathrm{j}}\max \left(0,{A}_{{\mathrm{n}}_{\mathrm{i},\mathrm{j}}}\right)\right)\right) \end{align*}

where the outer sum runs over the 100 canopy layers and the inner sum over the sunlit ($sl$) and shaded ($sh$) leaves; ${\varLambda}_{\mathrm{i}}$ is the leaf area (m${\kern0em }^2$) of the target tree in canopy layer $i$; ${f}_{\mathrm{i},\mathrm{sl}}=1-{f}_{\mathrm{i},\mathrm{sh}}$ is the sunlit fraction of leaves in canopy layer $i$; and ${A}_{{\mathrm{n}}_{\mathrm{i},\mathrm{j}}}$ and ${\Delta}_{\mathrm{i},\mathrm{j}}$ are the sunlit/shaded leaf net assimilation ($\mu$mol m${\kern0em }^{-2}$(leaf) s${\kern0em }^{-1}$) and ${\Delta}^{13}\mathrm{C}$ or ${\Delta}^{18}$O${\kern0em }_{\mathrm{lw}}$ (‰), respectively, corresponding to the plant type of the target tree in canopy layer $i$. The leaf-level ${\Delta}^{13}\mathrm{C}$ and ${\Delta}^{18}$O${\kern0em }_{\mathrm{lw}}$ were temporally aggregated to 24-day window moving average to reflect the cell maturation period (see Formation time of measured ${\mathrm{\delta}}^{13}\mathrm{C}$ and ${\mathrm{\delta}}^{18}\mathrm{O}$).

#### Model runs, parametrization and evaluation

We ran the model from September 2009 until the end of 2020 using the LAD profiles derived for the 10 plots surrounding the target trees, i.e., 15 stands in total as the thinned plots were run for both pre- and post-thinning conditions (Figure S1 available as Supplementary data at *Tree Physiology Online*). We note that we did not change the LAD profile in the simulation once it was initialized which might affect the results. We adopted the other model parameters from Leppä et al. (2020), who used the pyAPES model for the same site. In addition to stand characteristics, parameters differing between the control and the thinned area included field layer vegetation LAI and coverage of moss and litter on the forest floor (see Leppä et al. 2020). Leppä et al. (2020) obtained good agreement between measured and modeled 30-min resolution ecosystem energy and water fluxes and gross primary production for growing seasons of the pre-thinning period, 2010–15. The ${R}^2$ were 0.98, 0.84, 0.60 and 0.77 for net radiation, sensible heat flux, latent heat flux and gross primary production, respectively. The pyAPES microclimatic predictions have been further evaluated in Launiainen et al. (2015), who showed the model is able to predict the measured within-canopy radiation profiles, CO_2_, H_2_O and temperature gradients and soil moisture profile in a boreal pine forest.

Based on model results, we evaluated the differences in micrometeorological conditions within the canopy between the control plots, and the harvest plots before and after thinning. The vertical profiles of micrometeorological variables were calculated as averages of daytime (sun above horizon) conditions during growing seasons in order to focus of the relevant periods for wood formation. We defined the growing season from the wood formation periods with the CASSIA model (see section Sample preparation and isotopic analysis).

Furthermore, we evaluated the tree-level ${\Delta}^{13}\mathrm{C}$ and ${\Delta}^{18}$O${\kern0em }_{\mathrm{lw}}$ by upscaling the modeled leaf-level ${\Delta}^{13}$C and ${\Delta}^{18}$O${\kern0em }_{\mathrm{lw}}$ to the level of target trees following Eq. (9) with the different variations of Eqs (6) and (7), respectively. To compare modeled ${\Delta}^{13}\mathrm{C}$and ${\Delta}^{18}$O${\kern0em }_{\mathrm{lw}}$ against corresponding values derived from the measured tree-ring isotopic ratios, we calculated assimilation-weighted 24-day rolling means and means for EW and LW periods (see section Sample preparation and isotopic analysis) using results from the growing season and from the daytime.

#### Sensitivity analysis of leaf-level processes

To identify main abiotic drivers and key processes behind the changes in ${\Delta}^{13}\mathrm{C}$, we utilized a one-at-a-time (OAT) local sensitivity analysis (LSA) to study how sensitive the modeled ${\Delta}^{13}\mathrm{C}$ is to changes in photosynthesis and water use parameters. We ran the LSA only for the needle–gas exchange module of the pyAPES model. We limited the OAT analysis to this module only in order to reduce the computational burden for running millions of simulations.

For the meteorological variables, we performed the OAT analysis by first setting every meteorological variable to the median value from the vertically modeled microclimate. All other model parameters were set to the same value they were on the pyAPES simulations described above (section Model runs, parametrization and evaluation). We then varied one meteorological variable from its minimum simulated value to the maximum value and recorded ${\Delta}^{13}\mathrm{C}$.

For the photosynthesis and water use parameters, we performed the OAT analysis two different ways. First, we set all the meteorological variables to their median values and then changed either RH, PAR or needle temperature. For this new perturbed condition, we performed three simulations: one with the original parameter values, one where a single photosynthesis or water use parameter was decreased 10% and one where the parameter was increased by 10%. The sensitivity here was quantified as the change in ${\Delta}^{13}\mathrm{C}$


(10) \begin{equation*} \Delta \left({\Delta}^{13}\mathrm{C}\right)={\Delta}^{13}\mathrm{C}\left({\mathbf{x}}_{\mathbf{i}}\right)-{\Delta}^{13}\mathrm{C}\left({\mathbf{x}}_{\mathbf{p}}\right) \end{equation*}

where ${\text{x}}_{\text{i}}$ are the initial input variables where all the meteorological variables, except one, are at their median value and the photosynthesis or water use parameters are at their original values. ${\text{x}}_{\text{p}}$ are the input variables where a single parameter has been perturbed.

Second, we calculated how ${\Delta}^{13}\mathrm{C}$ changes when the parameters are perturbed by $\pm 10\%$ as above but in this case the meteorological conditions were taken from the simulated meteorology on the selection harvest plot after the harvest during the daytimes of the growing season and from every layer in the model where $\mathrm{LA}{\mathrm{D}}_{\mathrm{spruce}}>0\ {\mathrm{m}}^2{\mathrm{m}}^{-3}$ (for LAD of the studied plots see Figure S1 available as Supplementary data at *Tree Physiology Online*).

## Results

### *Comparison of predicted and observed* ${\Delta}^{13}C$

**Figure 1:**
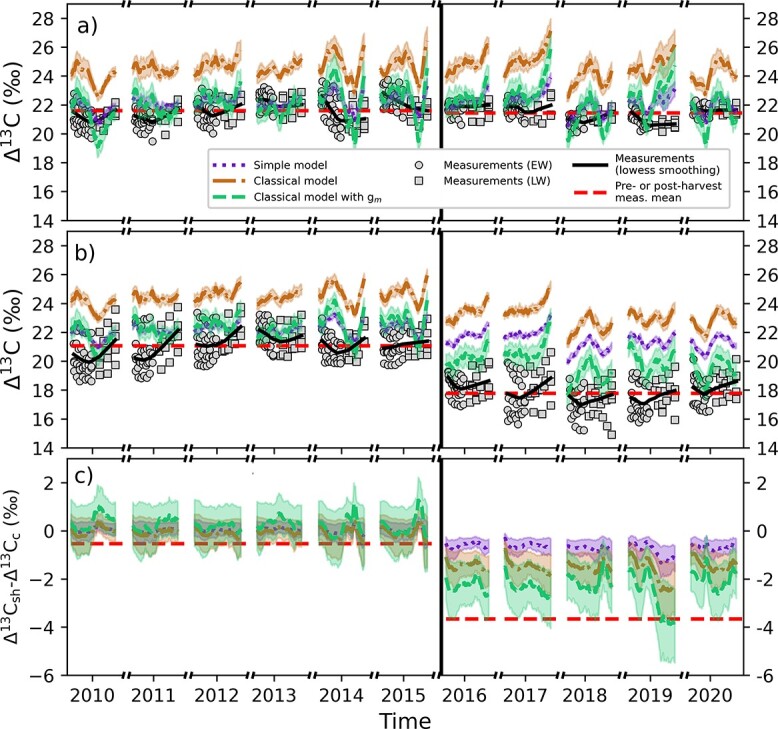
Modeled and measured ${\Delta}^{13}\mathrm{C}$ for (a) control plot, (b) selection harvest plot and (c) difference in ${\Delta}^{13}\mathrm{C}$ between the plots (sh = selection harvest, c = control). The data consist of the daytimes of growing season. Model simulations correspond to assimilation-weighted 24-day rolling mean in the corresponding plot and the patched area marks one standard deviation from the mean between different stands.   Model results show the three different ${\Delta}^{13}\mathrm{C}$ models described in section Leaf-level isotope discrimination models. The classical model with *g*_m_ was run with *g*_m_ = 0.1 mol m^−2^ (leaf) s^−1^. Circles (EW) and squares (LW) show the measured ${\Delta}^{13}\mathrm{C}$ of the target trees and the horizontal black lines show smoothed trend of the measurements. The red horizontal dashed lines show the mean of measured ${\Delta}^{13}\mathrm{C}$ in pre- and post-harvest periods. The vertical black line divides the data to pre- (left) and post-harvest (right) periods.

The measured ${\Delta}^{13}\mathrm{C}$ on the control plot remained fairly constant between pre- and post-harvest years (Figure 1a and Table 1). The mean ${\Delta}^{13}\mathrm{C}$ was 21.6 $\pm$ 0.9‰ (mean $\pm$ standard deviation) on the control plot before the selection harvest and 21.4 $\pm$ 0.6‰ after the selection harvest. On the selection harvest plot, the mean ${\Delta}^{13}\mathrm{C}$ showed a clear decrease already on the same year following the harvest (2016; Figure 1b). Before the harvest ${\Delta}^{13}\mathrm{C}$ was on average 21.1 $\pm$ 1.1‰ and after the harvest 17.7 $\pm$ 1.1‰.

**Table 1:** Coefficient of determination (*R*^2^) between the modeled and measured ${\Delta}^{13}\mathrm{C}$ and ${\Delta}^{18}{\mathrm{O}}_{\mathrm{lw}}$ and the difference between mean pre- (2010–15) and post-harvest (2016–20) ${\Delta}^{13}\mathrm{C}\ \mathrm{and}\ {\Delta}^{18}{\mathrm{O}}_{\mathrm{lw}}$ (i.e., the harvest response). Negative harvest response values mean decrease and positive mean increase in the corresponding discrimination after the selection harvest. The *R*^2^ value is calculated from yearly formation period (EW/LW) means for control and harvest plots and for the whole studied period of 2010–20.

Model	*R* ^2^		Difference between pre- and post-harvest years (‰)	
	Control	Selection harvest	Control	Selection harvest
${\Delta}^{13}\mathrm{C}$ : simple	0.06	0.56	0.0 $\pm$ 0.4	−0.7 $\pm$ 0.5
${\Delta}^{13}\mathrm{C}$ : classical	0.04	0.75	0.0 $\pm$ 0.6	−1.4 $\pm$ 0.5
${\Delta}^{13}\mathrm{C}$ : classical with *g*_m_ = 0.1 mol m^−2^ s^−1^	0.03	0.79	−0.2 $\pm$ 0.9	−2.4 $\pm$ 0.7
${\Delta}^{13}\mathrm{C}$ : measurements	—	—	−0.2 $\pm$ 0.7	−3.3 $\pm$ 1.6
${\Delta}^{18}{\mathrm{O}}_{\mathrm{lw}}$ : Craig–Gordon	0.67	0.46	1.3 $\pm$ 2.0	1.4 $\pm$ 2.1
${\Delta}^{18}{\mathrm{O}}_{\mathrm{lw}}$ : Péclet	0.68	0.43	1.3 $\pm$ 1.8	1.2 $\pm$ 1.9
${\Delta}^{18}{\mathrm{O}}_{\mathrm{lw}}$ : measurements	—	—	1.2 $\pm$ 0.9	1.6 $\pm$ 1.5

The modeled harvest response (how much ${\Delta}^{13}\mathrm{C}$ changed between pre- and post-harvest periods) was different for the different ${\Delta}^{13}\mathrm{C}$ models (Figure 1c). The simple model predicted the weakest harvest response and the classical model with ${g}_{\mathrm{m}}$ being the strongest. The absolute value of the change in ${\Delta}^{13}\mathrm{C}$ between pre- and post-harvest years were 0.7 $\pm$ 0.5‰, 1.4 $\pm$ 0.5‰ and 2.4 $\pm$ 0.7‰ for simple, classical and classical model with ${g}_{\mathrm{m}}$, respectively. All the ${\Delta}^{13}$C models showed smaller harvest response compared with the measured harvest response of 3.3 $\pm$ 1.6‰.

In the control plot, the modeled ${\Delta}^{13}\mathrm{C}$ was not able to capture much of the variation of measured ${\Delta}^{13}\mathrm{C}$ on the temporal resolution of EW/LW (Table 1; Figure S2 available as Supplementary data at *Tree Physiology Online*) likely because of only a small variability between years and the uncertainties in dating the ${\Delta}^{13}\mathrm{C}\ \mathrm{measurements}$. In the partial harvest plot, the variation of ${\Delta}^{13}\mathrm{C}$ was higher because of harvest-induced changes, which resulted in higher model fit compared with the control area (Figure S2 available as Supplementary data at *Tree Physiology Online*). The model–measurement match improved with increasing model complexity and the coefficient of determination (*R*^2^) was 0.79 for the classical model with ${g}_{\mathrm{m}}$. The mean vertical profiles of the ${\Delta}^{13}\mathrm{C}$ models shown in Figure S3 available as Supplementary data at *Tree Physiology Online* reveal that leaf-level ${\Delta}^{13}\mathrm{C}$ is higher at the bottom of the crown and lower at the top. The difference between minimum and maximum ${\Delta}^{13}\mathrm{C}$ are highest for the classical model with ${g}_{\mathrm{m}}$ and smallest for the simple model. The difference is higher for the control plot than for the selection harvest plot and for the pre-harvest period compared with the post-harvest period on the selection harvest plot.

### *Comparison of predicted and observed* ${\Delta}^{18}{O}_{lw}$

**Figure 2:**
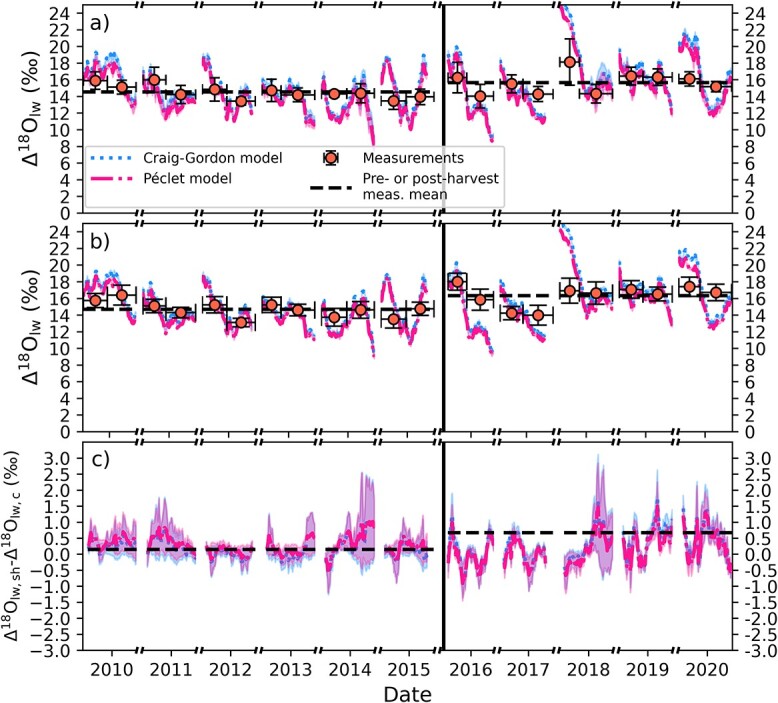
Modeled and measured leaf water ^18^O enrichment, ${\Delta}^{18}{\mathrm{O}}_{\mathrm{lw}}$, for (a) control plot, (b) selection harvest plot and (c) difference in ${\Delta}^{18}{\mathrm{O}}_{\mathrm{lw}}$ between the plots (sh = selection harvest, c = control). The data consist of the daytimes of growing seasons. Model simulations correspond to average of assimilation-weighted 24-day rolling mean of the target trees in the corresponding plot and the lighter patches show one standard deviation between different stands. Model results show the two different ${\Delta}^{18}{\mathrm{O}}_{\mathrm{lw}}$ models described in section Leaf-level isotope discrimination models. Orange markers show the average of the measured values of the target trees. For each year, there are two measurements from five trees per treatment (harvested vs control), one corresponding to EW and one to LW. The vertical error bars show the standard deviation of these measurements (*n* = 5), whereas the horizontal error bars show the start of the growing season and its middle point (for EW measurement points) or middle point of the growing season and its end (for LW measurement points). Horizontal dashed black lines show the mean measured ${\Delta}^{18}{\mathrm{O}}_{\mathrm{lw}}$ in pre- and post-harvest periods, whereas the black solid vertical line marks the division between pre (left)- and post-harvest (right) periods.

**Figure 3:**
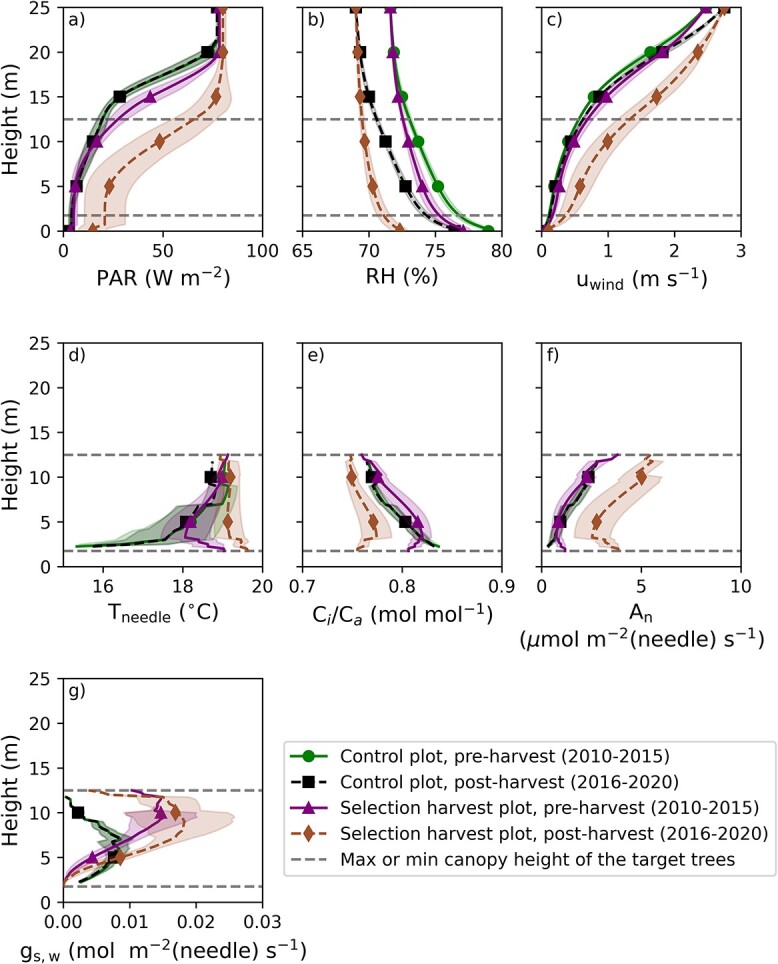
Modeled average vertical profiles for control and selection harvest plots from daytime of growing seasons during 2010–15 (control and selection harvest plot pre-harvest, green and magenta, respectively) and 2015–20 (control and selection harvest plot, post-harvest, black and brown, respectively). (a) PAR, (b) RH, (c) wind speed, (d) needle temperature, (e) leaf internal and atmospheric CO_2_ concentration ratio, (f) CO_2_ assimilation rate and (g) stomatal conductance for gaseous water. Marked lines show the mean of the variable and the shaded area the standard deviation of the variable between different stands. Black dashed horizontal lines show the range where the target trees have needles. Parameters in the second and third row (d–g) are weighted by the estimated CO_2_ assimilation rate of the target trees. Note that the mean PAR, RH and wind speed above the canopy (at 25 m height) differ between pre- and post-harvest, indicating different macroclimatic forcing between these periods.

After the harvest, the measured ${\Delta}^{18}{\mathrm{O}}_{\mathrm{lw}}$ on the selection harvest plot increased 1.6‰ on average compared with ${\Delta}^{18}{\mathrm{O}}_{\mathrm{lw}}$ in the post-harvest period (Figure 2). However, the measured ${\Delta}^{18}{\mathrm{O}}_{\mathrm{lw}}$ increased also on the control plot between pre- and post-harvest periods by 1.1‰ (Table 1). Based on statistical testing, the post-harvest ${\Delta}^{18}{\mathrm{O}}_{\mathrm{lw}}$ values on both plots are distributed approximately similarly (two-sample Kolmogorov–Smirnov test, *P*-value = 0.05). These results indicate that there was no clear harvest response in ${\Delta}^{18}{\mathrm{O}}_{\mathrm{lw}}$, but that the majority of the increased ${\Delta}^{18}{\mathrm{O}}_{\mathrm{lw}}$ after the harvest observed in both plots comes from the variability in meteorological conditions between pre- and post-harvest years. The RH was on average smaller (Figure 3b) during post-harvest years and the wind speed was higher (Figure 3c), which both have an increasing effect on ${\Delta}^{18}{\mathrm{O}}_{\mathrm{lw}}$. Similar to the measurements, the modeled ${\Delta}^{18}{\mathrm{O}}_{\mathrm{lw}}$ showed close to no change because of the harvest (Figure 2c). The modeled ${\Delta}^{18}{\mathrm{O}}_{\mathrm{lw}}$ increased 1.4‰ (Craig–Gordon model) and 1.2‰ (Péclet model) from pre- to post-harvest period for the selection harvest plot, whereas the increase was 1.3‰ for both models on the control plot. The similar response on both plots is in line with the measurements, supporting the conclusion that the main driver for the change was the less humid posttreatment years.

The ${\Delta}^{18}{\mathrm{O}}_{\mathrm{lw}}$ models explained 43–68% of the variability of measured ${\Delta}^{18}{\mathrm{O}}_{\mathrm{lw}}$ at EW/LW resolution (Table 1 and Figure S4 available as Supplementary data at *Tree Physiology Online*). The Péclet model produced slightly better match (*R*^2^ = 0.68 vs 0.67) on the control plot. On the contrary, the Craig–Gordon model outperformed marginally the Péclet model on the selection harvest plot (*R*^2^ = 0.46 vs. 0.43).

### *Drivers of the modeled* ${\Delta}^{13}C$

The modeled ${\Delta}^{13}\mathrm{C}$ is jointly determined by the microclimatic conditions and the responses of plant photosynthetic and water use parameters to harvest. First, the micrometeorological conditions above the canopy and the canopy structure determine, e.g., the PAR intensity and CO${\kern0em }_{{2}}$ availability for photosynthesis at each canopy layer. Second, plant photosynthesis and water- use parameters in the model determine how well the trees can utilize the available resources. The modeled change in ${\Delta}^{13}\mathrm{C}$ in Figure 1 shows how changes in micrometeorology are reflected to changes in ${\Delta}^{13}\mathrm{C}$ if no physiological stress or plant acclimation to harvest takes place (photosynthesis or water-use parameters stay the same).

#### Micrometeorological drivers

After the harvest, PAR and wind speed clearly increased in the lower canopy because of the removal of large fraction of LAD (Figure 3a and c). Relative humidity (Figure 3b) decreased both because of the smaller LAD and from changes in macroclimatic conditions between pre- and post-harvest years. The average needle temperature increased slightly (ca 1 ${\kern0em }^{\circ }$C) in the lower canopy because of the increased radiation available to the needles (Figure 3d).

As a result of the increased PAR and decreased RH, the average ${C}_{\mathrm{i}}/{C}_{\mathrm{a}}$ ratio decreased (Figure 3e). The decrease was most notable at the bottom of the canopy where the average ${C}_{\mathrm{i}}/{C}_{\mathrm{a}}$ ratios are 0.80 and 0.75 for the pre- and post-harvest periods, respectively.

Figure 3f and g shows the average changes in ${A}_{\mathrm{n}}$ and stomatal conductance for water (${g}_{\mathrm{s},\mathrm{w}}$). After the harvest, ${A}_{\mathrm{n}}$ of the spruces clearly increased because of improved light availability in the whole canopy, whereas ${g}_{\mathrm{s},\mathrm{w}}$ increased only slightly because of decreased RH (increased vapor pressure deficit), which decreases ${g}_{\mathrm{s},\mathrm{w}}$ and counterbalances the increase in ${g}_{\mathrm{s},\mathrm{w}}$ because of increased ${A}_{\mathrm{n}}$. Figure 3 shows also vertical profiles for the control plot. Overall, the canopy profiles are similar between the control and the selection harvest plots prior to the harvest indicating that the micrometeorological conditions were comparable between the selection harvest plot before the harvest and the control plot except for RH, ${g}_{\mathrm{s},\mathrm{w}}$ and ${T}_{\mathrm{needle}}$ in the bottom of the canopy.

We performed an OAT sensitivity analysis to study which forcing variables affect the ${\Delta}^{13}\mathrm{C}$ the most (Figure S5 available as Supplementary data at *Tree Physiology Online*). The analysis revealed that modeled ${\Delta}^{13}\mathrm{C}$ is most sensitive to RH, PAR and ${T}_{\mathrm{needle}}$, whereas the wind speed and ambient CO${\kern0em }_2$ concentration showed only minor sensitivity.

**Figure 4:**
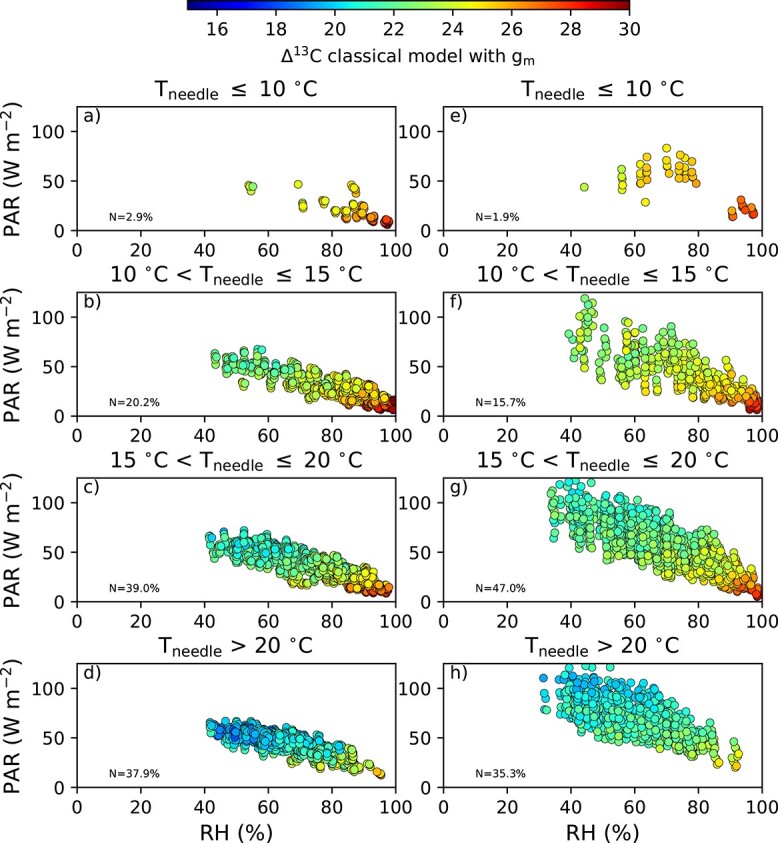
Average daily needle temperature (*T*_needle_), PAR and RH available for the spruce trees from every growing season between 2010–15 (pre-harvest, a–d) and 2016–20 (post-harvest, e–h). The daily average is calculated only for daytime. The color of the circles shows the value of classical ${\Delta}^{13}\mathrm{C}$ model with *g*_m_.

Figure 4 shows the daily average RH and PAR binned to different ${T}_{\mathrm{needle}}$ intervals on the selection harvest plot. The left hand side column (Figure 4a–d) corresponds to preharvest and the right hand side column (Figure 4e–h) to post-harvest years.

In the post-harvest conditions, the slope of the PAR as a function of RH is steeper than in the pre-harvest conditions. The steeper slope indicates increased PAR intensity that increases CO${\kern0em }_2$ assimilation in the model and, consequently, lowers ${\Delta}^{13}\mathrm{C}.$ Decrease in RH implies decreased ${\Delta}^{13}\mathrm{C}$ as the highest ${\Delta}^{13}\mathrm{C}$values are associated with high RH values. Figure 4 reveals where the change in average canopy profiles originates: after the harvest the days when the average RH $\ge 90\%$ is remarkably lower than before the harvest (13 vs 9$\%$ of all the days before and after harvest, respectively). The average RH increases the most in interval $40\%\le \mathrm{RH}\le 50\%$ in post-harvest period (6 vs 12$\%$).

#### Photosynthesis and water-use parameters

**Figure 5:**
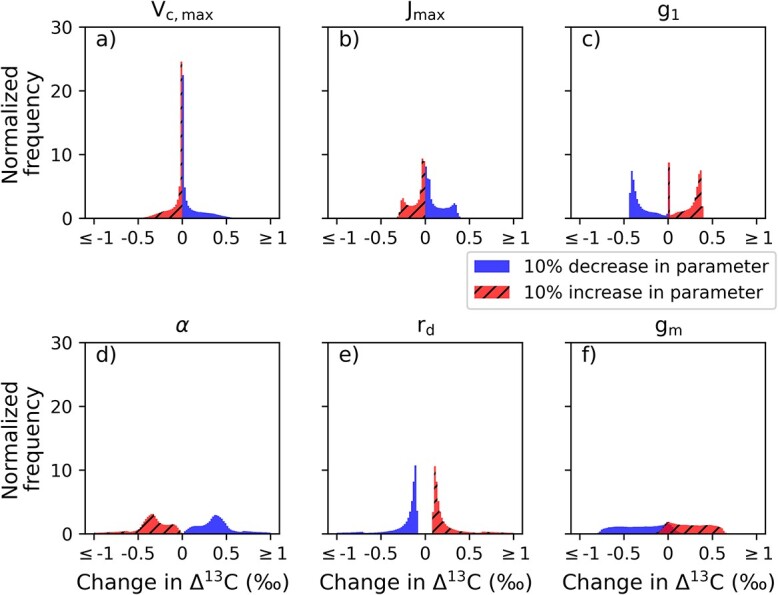
Sensitivity of the modeled ${\Delta}^{13}\mathrm{C}$ to photosynthesis and water-use parameters in post-harvest conditions: (a) maximum carboxylation velocity *V*_c,max_ and (b) maximum electron transport rate *J*_max_, (c) stomatal conductance parameter *g*_1_, (d) quantum yield parameter α, (e) mitochondrial (dark) respiration rate, *r*_d_ and (f) mesophyll conductance *g*_m_. Each parameter is perturbed by either decreasing or increasing its original value by 10%. The distributions are calculated in each environmental condition in the post-harvest growing seasons daytime by first calculating the original ${\Delta}^{13}\mathrm{C}$ then perturbing the parameter and subtracting the perturbed ${\Delta}^{13}\mathrm{C}$ from the original one. The horizontal axis shows how many units (‰) ${\Delta}^{13}\mathrm{C}$ changes.

Because the classical model with ${g}_{\mathrm{m}}$ (full Eq. (6)) was able to best capture the harvest response, the subsequent sensitivity analyses were conducted for this model only. Figure 5 shows the sensitivity of ${\Delta}^{13}\mathrm{C}$ to $\pm 10\%$ perturbations in the key photosynthesis and water use parameters in the model, calculated with the second method described in section Sensitivity analysis of leaf-level processes.

In the ambient conditions observed at Lettosuo after the harvest, changing the maximum carboxylation rate (${V}_{\mathrm{c},\max }$) or the maximum electron transport rate (${J}_{\mathrm{max}}$) did not substantially change ${\Delta}^{13}\mathrm{C}$, implying that any increase in photosynthetic CO_2_ demand is compensated by near-proportional increase in ${g}_{\mathrm{s}}$ in the model leading to no changes in ${C}_{\mathrm{i}}/{C}_{\mathrm{a}}$ ratio. The model sensitivity is clearly highest for the ${g}_1$ parameter (Eq. (5)) and for the mesophyll conductance ${g}_{\mathrm{m}}$ directly affecting the efficiency of CO_2_ diffusion from leaf surface to reaction sites in chloroplasts.

The ${\Delta}^{13}\mathrm{C}$ was also sensitive to changes in quantum yield parameter ($\alpha$) and dark respiration rate (${r}_d$). However, Figure S6 available as Supplementary data at *Tree Physiology Online* shows that the sensitivity of ${\Delta}^{13}\mathrm{C}$ for these parameters is highest during low PAR intensity. Because the selection harvest increases the PAR intensity, it is unlikely that changing these parameters would yield lower modeled ${\Delta}^{13}\mathrm{C}$ and better match for the measured harvest response at Lettosuo.

**Figure 6:**
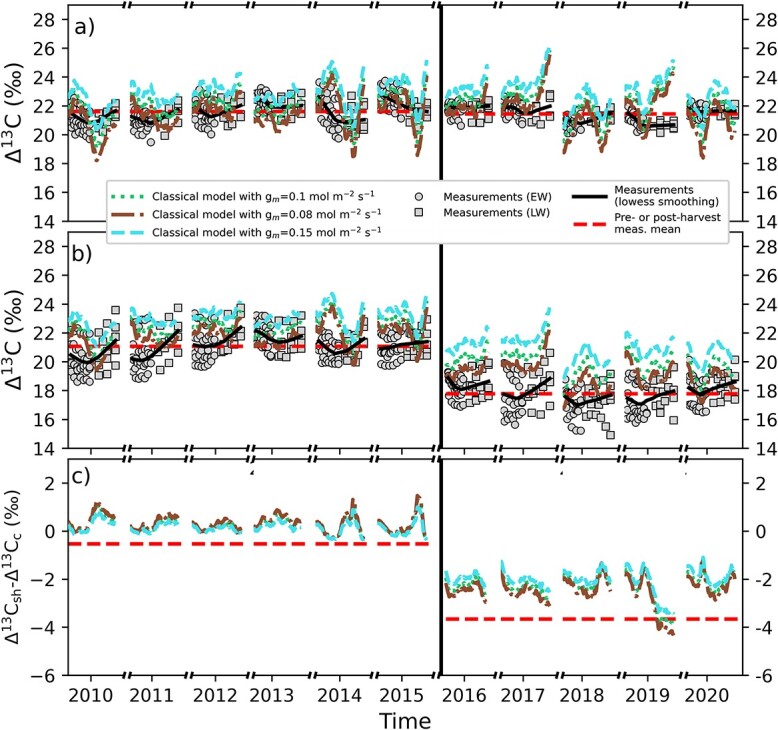
Sensitivity of modeled ${\Delta}^{13}\mathrm{C}$ to mesophyll conductance (*g*_m_) for (a) control plot, (b) selection harvest plot and (c) difference in ${\Delta}^{13}\mathrm{C}$between the plots (sh = selection harvest, c = control). The data consist of the daytimes of growing season. Model simulations correspond to assimilation-weighted 24-day rolling mean in the corresponding plot. Different model simulations correspond to pyAPES simulations with different *g*_m_. Gray circles (EW) and squares (LW) show the measured ${\Delta}^{13}\mathrm{C}$ of the target trees and the horizontal black lines show smoothed trend of the measurements. The red dashed lines show the mean of measured ${\Delta}^{13}\mathrm{C}$ in pre- and post-harvest periods. The vertical black line divides the data to pre- (left) and post-harvest (right) periods.

Because ${\Delta}^{13}\mathrm{C}$ showed high sensitivity for ${g}_{\mathrm{m}}$, we ran two additional simulations with pyAPES where the ${g}_{\mathrm{m}}$ was first decreased to 0.08 $\mathrm{mol}\ {\mathrm{m}}^{-2}\ {\mathrm{s}}^{-1}$ (lower limit for confers in Flexas et al. 2012) and another where it was increased to $0.15\ \mathrm{mol}\ {\mathrm{m}}^{-2}\ {\mathrm{s}}^{-1}$. The effect of changing ${g}_{\mathrm{m}}$ to ${\Delta}^{13}\mathrm{C}$ is shown Figure 6. When ${g}_{\mathrm{m}}$ was decreased the harvest response improves to 2.6 $\pm$ 0.7‰ and when it was increased the response decreases to 2.1 $\pm$ 0.7‰. The same improvement in the harvest response (2.6‰) was obtained when ${g}_1$ was decreased by 20% (results not shown). An even higher improvement (3.1‰) would result from the smaller ${g}_1$ occurring only during post-harvest representing a physiological adjustment of the previously suppressed trees. We note that changing ${g}_1$ alone would lead to changes in the fit against ecosystem level fluxes reported in Leppä et al. (2020).

## Discussion

The decrease in the measured tree-ring ${\Delta}^{13}\mathrm{C}$ in previously suppressed Norway spruce trees on the selection harvest plot (Figure 1b) implies that the ${A}_{\mathrm{n}}/{g}_{\mathrm{s}}$ ratio increased after the harvest. The measured tree-ring ${\Delta}^{18}{\mathrm{O}}_{\mathrm{lw}}$ (Figure 2) showed similar changes on the control and selection harvest plots, which indicates the change was not caused by the harvest. Instead, the changes in ${\Delta}^{18}{\mathrm{O}}_{\mathrm{lw}}$ result mostly from changes in environmental forcing between pre- and post-harvest years (e.g., RH, Figure 3b). The process-model simulated similar responses to harvest, i.e., a decrease in ${\Delta}^{13}\mathrm{C}$ (albeit smaller than the observed) and no change in ${\Delta}^{18}{\mathrm{O}}_{\mathrm{lw}}$ (Figures 1c and 2c). At the same time, the model predicted that leaf and tree-level ${A}_{\mathrm{n}}$increased relatively more than ${g}_{\mathrm{s}}$ after the harvest, but that ${g}_{\mathrm{s}}$ still did increase (Figure 3f and g). While the observed ${\Delta}^{13}\mathrm{C}$ decrease led to the conclusion of a more increased ${A}_{\mathrm{n}}$relative to ${g}_{\mathrm{s}}$, we were not able to make the conclusion of increased ${g}_{\mathrm{s}}$ from the measurement based ${\Delta}^{18}{\mathrm{O}}_{\mathrm{lw}}$ (expecting decrease instead of no change/small increase; Figure 2c). This is because in this case, the correlation between ${g}_{\mathrm{s}}$ and RH is interfered by the strong change in ${A}_{\mathrm{n}}$ in response to harvest (Eq. (5); Roden and Farquhar 2012, Roden and Siegwolf 2012).

The modeled ${\Delta}^{13}\mathrm{C}$ decreased with all the three studied ${\Delta}^{13}\mathrm{C}$ models because of the harvest. The light availability (PAR intensity) and ${T}_{\mathrm{needle}}$ were predicted to increase because of the harvest, whereas the observed RH decrease was caused by different meteorological conditions (weather) as well as the selection harvest. All these changes contribute to the decrease in the modeled ${\Delta}^{13}\mathrm{C}$ (Figures 3 and 4). The models, however, predicted smaller decrease in ${\Delta}^{13}\mathrm{C}$ than what was observed (Figure 1 and Table 1).

Similar comparisons of modeled and measured isotopic harvest responses are scarce. Giuggiola et al. (2016) applied the MuSICA model (Ogée et al. 2003) to study thinning effects on Scots pine (*P. sylvestris*) on a xeric site. They found a good agreement between modeled and measured ${\delta}^{13}\mathrm{C}$ and ${\Delta}^{18}{\mathrm{O}}_{\mathrm{lw}}$. In our study, the modeled ${\Delta}^{18}{\mathrm{O}}_{\mathrm{lw}}$ matched the measurements well (Figure 2 and Figure S4 available as Supplementary data at *Tree Physiology Online*). We did not see a clear improvement in modeling ${\Delta}^{18}{\mathrm{O}}_{\mathrm{lw}}$ with the Péclet model compared with the traditional Craig–Gordon model. This result is in line with the suggestions of Cernusak et al. (2016) that over longer temporal scales and the further down the ^18^O is measured in the tree the variations because of the Péclet effect are smaller. The simulated ${\Delta}^{18}{\mathrm{O}}_{\mathrm{lw}}$ of the post-harvest years were in slightly better agreement with measurements at the control area compared with the harvested area (Figure S4 available as Supplementary data at *Tree Physiology Online*). It is possible that this is affected by a change in source water ${\delta}^{18}\mathrm{O}$ in response to harvest. A potential cause could be the decreased interception capacity of the canopy after harvest as canopy interception is known to modify the ${\delta}^{18}\mathrm{O}$ of throughfall compared with that of open-air precipitation (Kato et al. 2013).

Interpretations of thinning responses from ${\Delta}^{13}\mathrm{C}$ strongly rely on the relationship between ${\Delta}^{13}\mathrm{C}$ and ${A}_{\mathrm{n}}/{g}_{\mathrm{s}}$, despite the recent literature showing that discrimination processes may be more complicated in certain conditions and species. For example, when the ratio of mitochondrial respiration against net assimilation (${r}_{\mathrm{d}}/{A}_{\mathrm{n}}$) is high, discrimination by respiration may be more important than that of carboxylation (Busch et al. 2020). This may be relevant in dense canopies for suppressed trees with low ${A}_{\mathrm{n}}$. Our results show that the simple model performs comparably to the classical model with ${g}_{\mathrm{m}}$ on the control plot and selection harvest plot before the harvest. The fact that the simple model performed well on these plots shows that for the suppressed spruce trees it is the ${A}_{\mathrm{n}}/{g}_{\mathrm{s}}$ ratio that is the most relevant to ${\Delta}^{13}\mathrm{C}$.

Bickford et al. (2010) found similar to us that the simple model can produce better match to the measurements compared with more complex models. Our results also show that in the control plot and in the selection harvest plot prior to the harvest the simple model produces comparable results to the measurements. The simple model and its use in interpreting measured ${\Delta}^{13}\mathrm{C}$ were also discussed recently in Ubierna et al. (2022), where the authors show that the fractionation coefficient ${b}^{\prime }$ could be thought either as a free parameter that contains the combined effect of photosynthetic and non-photosynthetic fractionation when it is solved by fitting to measured ${\Delta}^{13}\mathrm{C}$ or as a mean to derive the ${A}_{\mathrm{n}}/{g}_{\mathrm{s}}$ ratio when ${b}^{\prime }$ is known. Moreover, Ubierna et al. (2022) emphasize that the ${\Delta}^{13}\mathrm{C}$ model should be chosen based on how dynamic the studied processes are and what kind of information is to be derived from the measured ${\Delta}^{13}\mathrm{C}$. For example, since the focus of our study is on process-level modeling of the ^13^C discrimination we should aim to apply a more detailed ${\Delta}^{13}\mathrm{C}$ model.

The role of mesophyll conductance on discrimination has been demonstrated for many species (Ubierna and Marshall 2011, Medlyn et al. 2017, Gimeno et al. 2021), including boreal coniferous species (Stangl et al. 2019). Because of the increased knowledge, studies implement leaf-level photosynthetic discrimination models of increasing complexity (Wingate et al. 2007, Stangl et al. 2019, Busch et al. 2020) but these more complex models have not been previously used in interpreting thinning response (Navarro-Cerrillo et al. 2016, Manrique-Alba et al. 2020). Our results highlight the importance of accounting for the above-mentioned processes when modeling the harvest response of previously suppressed Norway spruce trees. The results show that the classical model with ${g}_{\mathrm{m}}$ produced a harvest-response closest to the measurements (Figure 1 and Table 1). The importance of accounting for ${g}_{\mathrm{m}}$ was also recognized by Leppä et al. (2022), who found that accounting for diffusion of CO${\kern0em }_2$ in the mesophyll was necessary to predict the absolute level of ${\delta}^{13}\mathrm{C}$ in Scots pine needles.

All the ${\Delta}^{13}\mathrm{C}$ models produced a lower harvest response compared with the observations. There are multiple possible reasons for these deviations. The reasons can be divided to two groups: to processes that are not considered in the model and to uncertainty in model parameters.

It is possible that the processes omitted in the model cause the deviation between modeled and measured ${\Delta}^{13}\mathrm{C}$. For the carbon discrimination model (Eq. (6)), we did not consider any post-photosynthetic fractionations. While there are many physicochemical processes that are recognized as a possible source of down-stem fractionation (Gessler et al. 2014), detailed studies on the magnitude of these processes are still needed in order to implement them in carbon discrimination models. However, recent studies, which have examined these processes and their impact on tree-ring ${\delta}^{13}\mathrm{C}$ via a compound-specific and high spatial resolution approach for boreal *Larix gmelinii* (Rinne et al. 2015) and *P. sylvestris* (Tang et al. 2022, Rinne-Garmston, unpublished data), have indicated that the ^13^C-enrichment of resin extracted tree-rings relative to leaf sucrose is relatively small, varying from 0 to 1‰ for the five studied sites. For the studied conifers, no significant reliance on carbohydrate reserves, whose utilization would uncouple tree-ring ${\mathrm{\delta}}^{13}\mathrm{C}$ from instantaneous ambient environmental conditions, was detected in non-droughted conditions. If these results can be generalized also for spruces, it seems unlikely that the major isotopic offset (from 0.7 to 2.6‰) observed between the measured resin extracted wood ${\Delta}^{13}\mathrm{C}$ after the harvest (Figure 1) could be wholly explained by changes in the tree metabolic processes. It seems more probable that the post-photosynthetic fractionation could explain the systematic difference observed in ${\Delta}^{13}\mathrm{C}$ prior to the selection harvest and that the increase in model–measurement bias observed after the harvest is caused by the model parameters. This explanation is supported by Leppä et al. (2022), who found good agreement between modeled and measured ${\Delta}^{13}\mathrm{C}$ of Scots pine needles.

Regarding the model parameters, the deviation between modeled and measured ${\Delta}^{13}\mathrm{C}$ can originate either from the coefficients that determine the magnitude in discrimination (coefficients *a*–*e* in Eq. (6)) or from the model parameters used to calculate ${A}_{\mathrm{n}}$ and ${g}_{\mathrm{s}}$. Bickford et al. (2010) compared the measured and modeled ${\Delta}^{13}\mathrm{C}$ for junipers and found that reducing the fractionation because of carboxylation (coefficient b in Eq. (6)) caused a better model–measurement agreement than using the literature value. Since our modeled ${\Delta}^{13}\mathrm{C}$ is higher than the measured ${\Delta}^{13}\mathrm{C}$, lowering fractionation because of carboxylation in our study would also result to better model–measurement agreement.

We showed that perturbing the ${g}_1$ parameter of ${g}_{\mathrm{s}}$ (Eq. (5)) and the mesophyll conductance ${g}_{\mathrm{m}}$ showed the highest change in the modeled ${\Delta}^{13}\mathrm{C}$ (Figure 5) in the post-harvest environmental conditions. These results are in line with the calculations of Ubierna and Farquhar (2014), who showed that under similar *A* and ${g}_{\mathrm{m}}$ to our study it is the fractionation due to ${g}_{\mathrm{s}}$ and ${g}_{\mathrm{m}}$ that contribute the most to ${\Delta}^{13}\mathrm{C}$ after fractionation because of RuBisCO. The ${g}_1$ parameter is proportional to CO${\kern0em }_2$ compensation point ${\varGamma}^{\ast }$ and the marginal water cost of carbon gains parameter $\lambda$, which represents how many moles of water a tree is willing to lose to gain a mole of carbon (Medlyn et al. 2011). It seems plausible that the ${g}_1$ parameter could change as the needles acclimate to the new environment following the thinning. Also, Giuggiola et al. (2016) found that they needed to increase the stomatal slope parameter (similar to ${g}_1$ in our study) after a heavy thinning, whereas our sensitivity analysis suggests that ${g}_1$ would need to be decreased in order for the modeled ${\Delta}^{13}\mathrm{C}$ match better the measured values. This differing result is likely because of the site types: the site in Giuggiola et al. (2016) is xeric and the ${\delta}^{13}\mathrm{C}$ decreases (${\Delta}^{13}\mathrm{C}$ increases) following the thinning which suggests that ${g}_{\mathrm{s}}$ increases more relative to ${A}_{\mathrm{n}}$, and the opposite is true for our mesic site.

Similar to ${g}_1$, we used a constant value for ${g}_{\mathrm{m}}$ which also showed high sensitivity to ${\Delta}^{13}\mathrm{C}$ (Figure 5f). In their review, Flexas et al. (2012) discuss that ${g}_{\mathrm{m}}$ can change with, e.g., temperature, CO${\kern0em }_2$ availability, leaf nitrogen content, and along tree height or age of the needles. However, little is known about the functional forms of these dependencies especially in the case of Norway spruces. We further showed that decreasing ${g}_{\mathrm{m}}$ leads to better ${\Delta}^{13}\mathrm{C}$ harvest response (Figure 6) even though the measured harvest response was still higher than what was modeled with the lowest ${g}_{\mathrm{m}}$ reported in Flexas et al. (2012). This suggest ${g}_{\mathrm{m}}$ alone is not enough but that the previously suppressed trees may go through physiological adjustments decreasing ${g}_1$ in response to harvest.

Our OAT sensitivity analysis provides a simple estimate for the ${\Delta}^{13}\mathrm{C}$ sensitivity and does not consider any nonlinear effects or parameter interactions that may well be of importance for the ${\Delta}^{13}\mathrm{C}$ sensitivity in leaf gas exchange models. For example, Giuggiola et al. (2016) used different model parameters for studying the immediate (0–1 years after the thinning) and delayed effects (1–10 or more than 10 years after the thinning) of thinning on tree-ring isotopes. They found that when multiple parameters were changed, the stomatal slope parameter needed to be first increased from the immediate simulation to simulate the 1–10 years after the thinning and then to decrease the same parameter when studying the delayed effect after more than 10 years after the thinning. Therefore, utilizing global sensitivity analysis methods in the future (e.g., Song et al. 2015) would enable studying the parameters and their interactions holistically. For example, it might be that the parameters related to photosynthesis (e.g., ${V}_{\mathrm{c},\max },{J}_{\mathrm{max}}$) become more important when the other parameters are changed simultaneously. Besides model parameters, the modeling choices might also explain the model–measurement discrepancy. We modeled the carbon discrimination assuming that the plots are homogeneous in horizontal direction(s). Modeling the transport of energy and matter in three dimensions might provide ${\Delta}^{13}\mathrm{C}$ that better matches the measurements.

Regarding the hypothesis presented in the Introduction, we conclude the following. The selection harvest caused a decrease in ${\Delta}^{13}\mathrm{C}$ already on the first post-harvest growing season and the response persisted over the studied five post-harvest growing seasons. ${\Delta}^{18}{\mathrm{O}}_{\mathrm{lw}}$ did not show a clear change after the selection harvest. Together these findings indicate that the carbon assimilation rate ${A}_{\mathrm{n}}$ increased more than the stomatal conductance ${g}_{\mathrm{s}}$ after the harvest.

When the ${\Delta}^{13}\mathrm{C}$ was modeled with the pyAPES model, the changes in micrometeorological conditions because of the harvest explained ca 70% but not all the observed decrease in ${\Delta}^{13}\mathrm{C}$ (Hypothesis 1). The change in ${\Delta}^{13}\mathrm{C}$ was closest to the observed decline, when we explicitly accounted for the mesophyll conductance ${g}_{\mathrm{m}}$ (Hypothesis 2). Besides the lower harvest response, the modeled ${\Delta}^{13}\mathrm{C}$ was consistently higher than the observations at the selection harvest. These deviations can be caused partly from different fractionation coefficients or post-photosynthetic fractionation processes that we did not account for in the modeling. The main drivers of the predicted change in ${\Delta}^{13}\mathrm{C}$ were the PAR, RH and needle temperature (Hypothesis 3). Furthermore, the modeled ${\Delta}^{13}\mathrm{C}$ was the most sensitive to changes in the ${g}_1$ parameter used in calculating ${g}_{\mathrm{s}}$ and ${g}_{\mathrm{m}}$, which highlights the importance of accurate determination of these transport process parameters in the future (Hypothesis 4).

Combination of empirical data on tree-ring ${\Delta}^{13}\mathrm{C}$ and the soil–plant–atmosphere transfer model pyAPES for description of tree ecophysiology allowed us to evaluate major drivers for changes in the water-use strategy of trees after selection harvest. Increasing understanding of suppressed Norway spruce tree functioning and their carbon uptake and growth response following selection harvests is a prerequisite for evaluating climate change mitigation potential of CCF on drained peatlands. Advanced process-based models allow to study the carbon uptake and carbon allocation under drier air and higher light availability conditions after selection harvests. This is essential when future forest management guidelines are tailored and the intensity of the selection harvests is to be optimized based on site conditions, carbon uptake, environmental impacts and economical revenue.

## Supplementary Material

lettosuo_harvest_response_supplement_revision_tpad119

## Data Availability

LA-IRMS data analyzed in the isotope laboratory of Natural Resources Institute Finland and tree-ring width data of target trees are available in Zenodo: https://doi.org/10.5281/zenodo.5865404. HT-EA-IRMS data and the model simulations are available from the corresponding author.
