# Supplementary material for: Modeling the response of Norway spruce tree-ring carbon and oxygen isotopes to selection harvest on a drained peatland forest
Source: Tree Physiol. 2023 Sep 26;44(1):tpad119. doi: 10.1093/treephys/tpad119 (PMC10993295; doi:10.1093/treephys/tpad119)
Supplement: lettosuo_harvest_response_supplement_revision_tpad119 [file lettosuo_harvest_response_supplement_revision_tpad119.docx]

**Supplement for manuscript “Modelling the response of Norway spruce tree ring carbon and oxygen isotopes to selection harvest on a drained peatland forest” by Tikkasalo et. al.**


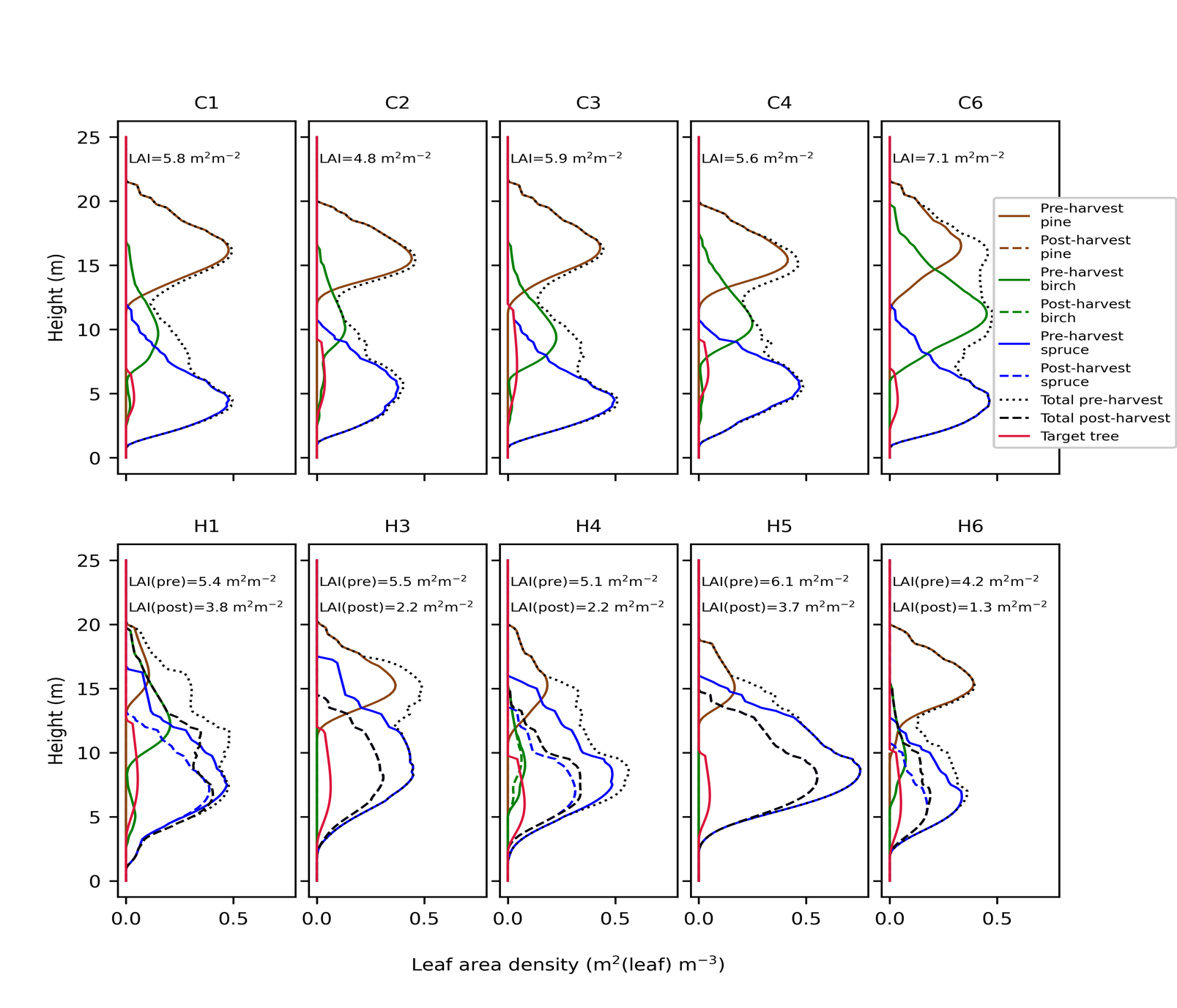


**Figure S1**: Vertical profiles of leaf area density (LAD) for each of the studied stand. Black dotted line shows the total LAD pre-harvest period and black dashed line for post-harvest period while brown, green and blue solid (for pre-harvest period) and dashed (for post-harvest period) lines show the LAD for pine, birch and spruce, respectively. Red solid lines show the LAD for the target trees from where the tree-ring samples were collected.

Figure S2: Modelled and measured yearly and seasonal $\Delta^{13}C$ values for control plot (a-c) and selection harvest plot (d-f). The column of the plot shows correspond to pyAPES simulations with the three different $\Delta^{13}C$ models described in the main text. The classical model with $g_{m}$ was run with $g_{m}=0.1 mol m^{-2}s^{-1}$. Color of the marker shows the year of the data point and the black dashed line is the 1:1 line and the solid line is the linear regression fit to the data. Circle markers are for the early wood formation period and square markers for the late wood period.


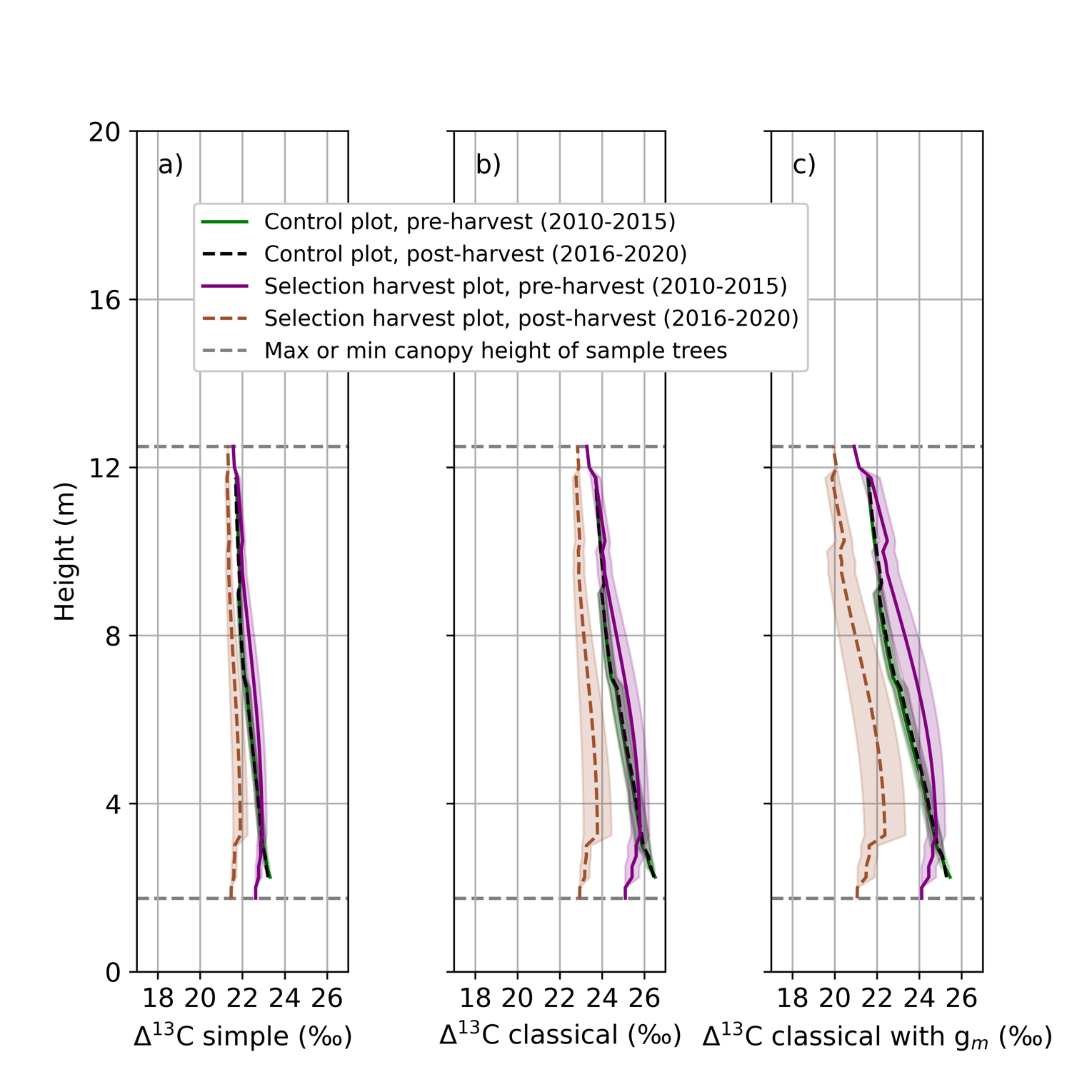


**Figure S3**: Modelled average vertical profiles of the three $\Delta^{13}C$ models used in this work. The profiles are calculated for control and selection harvest plots from daytime of growing seasons during 2010–2015 (control and selection harvest plot pre-harvest, green and magenta lines and patches) and 2016–2020 (control and selection harvest plot, post-harvest, black and brown lines and patches). a) simple $\Delta^{13}C$ model, b) classical $\Delta^{13}C$ model and c) classical $\Delta^{13}C$ model with mesophyll conductance $g_{m}$. Lines show the mean of the variable and the shaded area the standard deviation of the variable between different stands. Grey dashed horizontal lines show the range where the target trees have needles.

Figure S4: Modelled and measured yearly and seasonal $\Delta^{18}O_{lw}$ values for control plot (a-b) and selection harvest plot (c-d). The column of the plot corresponds to the pyAPES simulations with the two different $\Delta^{18}O$ models described in the main text. Color of the marker shows the year of the data point, the black dashed line is the 1:1 line and the solid line linear regression fit. Circle markers are for the early wood formation period and square markers for the late wood period.


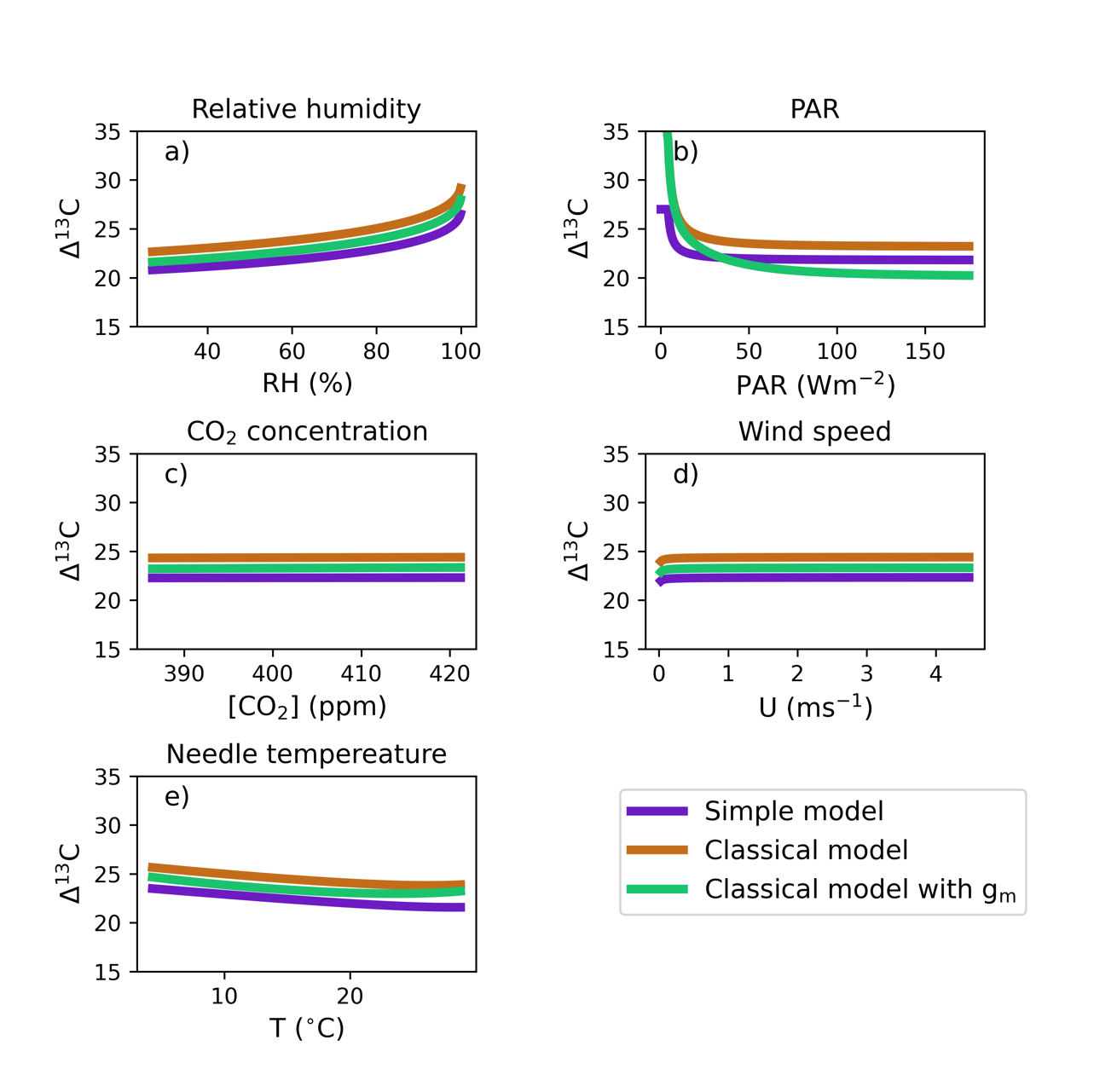


**Figure S5**: Local sensitivity analysis of the meteorological variables in the pyAPES model. The sensitivity analysis is done for the needle–gas exchange module of the model. The sensitivity is calculated by first setting every variable to its median value and then perturbing a single variable from its minimum to maximum value observed in the pyAPES simulations. The perturbed variables are a) relative humidity (RH), b) photosynthetically active radiation (PAR), c) CO${}_{2}$ concentration ([CO${}_{2}$]), d) wind speed (U) and e) needle temperature (T${}_{needle}$). The sensitivity is the highest for RH, PAR and T${}_{needle}$ while [CO${}_{2}$] and U show only minor sensitivity.


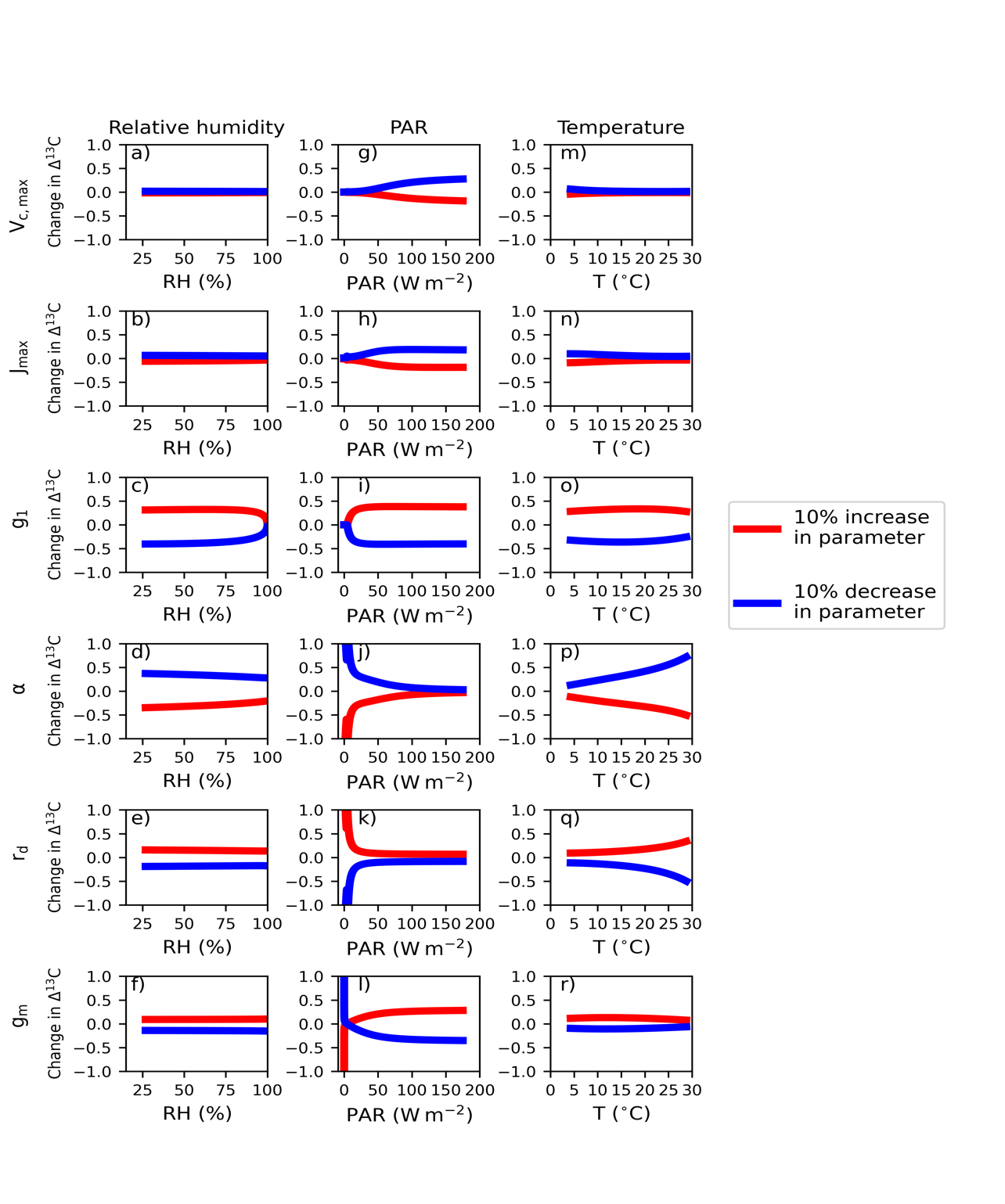


**Figure S6**: Local sensitivity analysis of the pyAPES model’s selected photosynthesis and water use parameters. The sensitivity is calculated using only the needle–gas exchange module of the pyAPES model by first setting the meteorological variables to their median values observed in the pyAPES simulations. Then a single meteorological variable (either relative humidity (RH), photosynthetically active radiation (PAR) or air temperature (T${}_{air}$) is allowed to vary from its minimum and maximum modelled value in the pyAPES simulations. At each meteorological condition the module is run three times: one with the original parameter values, one by decreasing a single parameter by 10$\%$ and one by increasing the parameter by 10$\%$. The vertical axis shows the change in $\Delta^{13}$C classical with $g_{m}$ relative to the simulation with the original parameter value. The studied parameters are from top to bottom row: maximum carboxylation velocity ($V_{c,max}$), maximum electron transport rate ($J_{max}$), $g_{1}$ parameter used to calculate stomatal conductance, quantum yield parameter $\alpha$, dark respiration rate $r_{d}$ and mesophyll conductance $g_{m}$.
